# Modeling and performance analysis of shuttle-based compact storage systems under parallel processing policy

**DOI:** 10.1371/journal.pone.0259773

**Published:** 2021-11-15

**Authors:** Lei Deng, Lei Chen, Jingjie Zhao, Ruimei Wang

**Affiliations:** 1 School of Information, Beijing Wuzi University, Beijing, China; 2 Beijing Municipal Tax Service, State Taxation Administration, Beijing, China; 3 College of Economics and Management, China Agricultural University, Beijing, China; Wageningen University, NETHERLANDS

## Abstract

Short response time for order processing is important for modern warehouses, which can be potentially achieved by adopting appropriate processing policy. The parallel processing policy have advantages in improving performance of many autonomous storage and retrieval systems. However, researchers tend to assume a sequential processing policy managing the movement of independent resources in shuttle-based compact storage systems. This paper models and analyses a single-tier of specialized shuttle-based compact storage systems under parallel processing policy. The system is modeled as a semi-open queueing network with class switching and the parallel movement of shuttles and the transfer car is modeled using a fork-join queueing network. The analytical model is validated against simulations and the results show our model can accurately estimate the system performance. Numerical experiments and a real case are carried out to compare the performance of parallel and sequential processing policies. The results suggest a critical transaction arrival rate and depth/width ratio, below which the sequential processing policy outperforms the parallel processing policy. However, the advantage of sequential processing policy is decreasing with the increasing of shuttle number, transaction arrival rate and depth/width ratio. The results also suggest an optimal depth/width ratio with a value of 1.75 for minimizing the expected throughput time in the real system. Given the current system configurations, the parallel processing policy should be considered when the number of shuttles is larger than 2 or the transaction arrival rate is larger than 24 per hour.

## 1 Introduction

**Fig 1:**
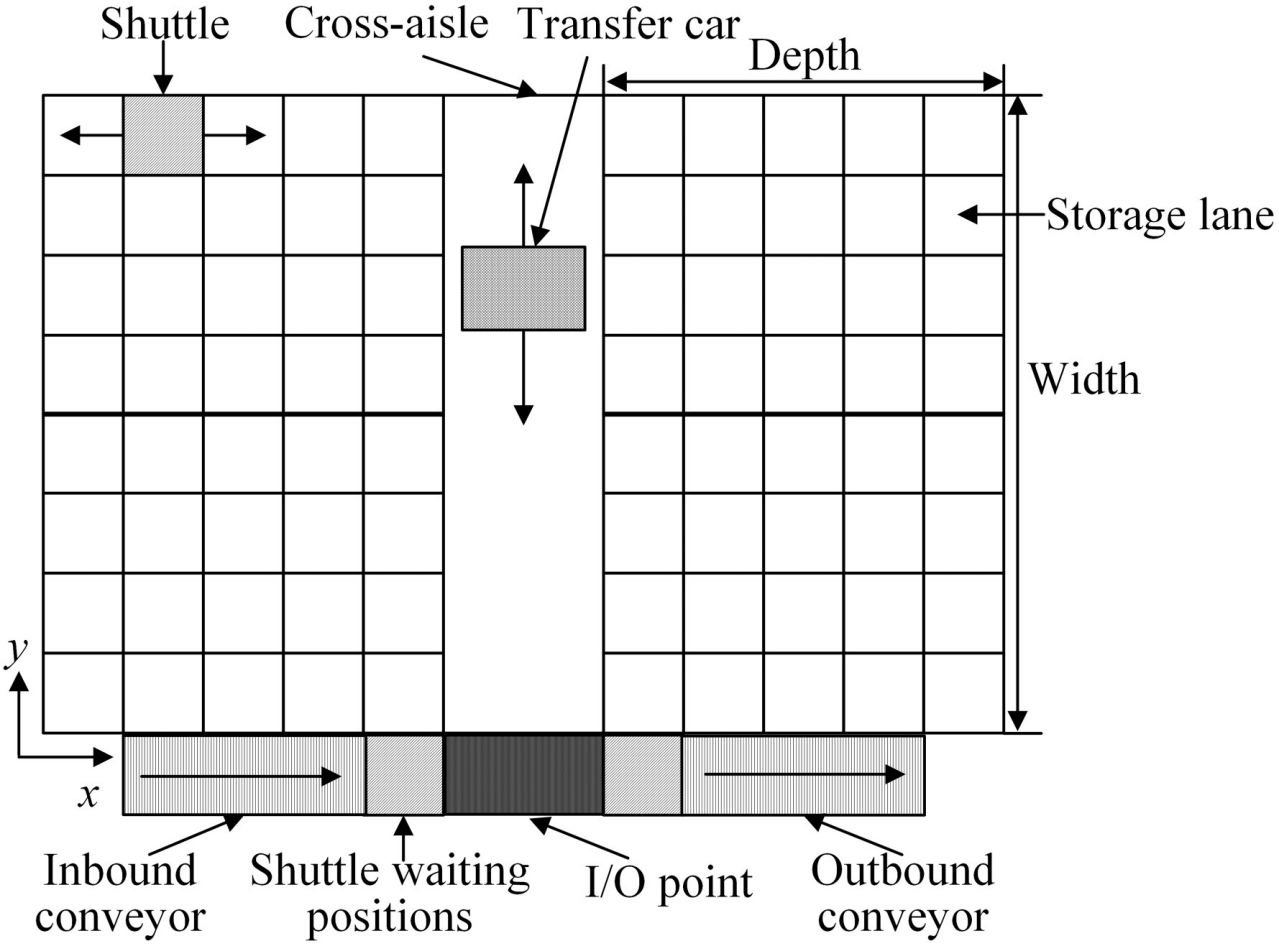
Top view of the shuttle-based compact storage system.

In recent years, customer demands for logistics and distribution become dynamic and keep changing, especially during COVID-19. This implies an increasing trend towards more service variety and shorter response times. As a new unit-load storage and retrieval system, shuttle-based compact storage systems combine the features of and are more cost-effective than autonomous vehicle-based storage systems and compact storage systems. Additionally, such systems are more time-saving and flexible, which means they have a shorter response time for storage or retrieval transactions and can change their throughput capacity by adding or removing shuttles [1, 2]. All these potential advantages of shuttle-based compact storage systems result to its growing popularity among and higher adoption by modern warehouses [3]. The shuttle-based compact storage systems consist of multiple tiers of multi-deep storage lanes. In such systems, the vertical movements moving loads across tiers are carried out using lifts and horizontal movements moving loads within the storage lanes are carried out using shuttles [1, 4]. The horizontal movements of a shuttle within cross-aisle, which is orthogonal to the storage lanes, can be performed either by a transfer car or by the shuttle itself. The shuttle that is transported to and from appropriate storage lanes by the transfer car is called specialized shuttle (Fig 1), while the one that can move both within the storage lanes and along cross-aisle is called generic shuttle. As our purpose is to model parallel movements of shuttles and transfer car, we only consider the systems with specialized shuttles.

In practice, the autonomous storage and retrieval systems are widely used and studied. Most of the literatures on this subject focus on the autonomous vehicle-based storage and retrieval systems (AVS/RS). In such systems, the vertical movements are performed by vehicles and horizontal movements are carried out by lifts. The most studied systems are characterized by multiple tiers of single- or double-deep storage racks. Malmborg [5] is the first to study AVS/RS systems. He builds a continuous markov chain model to describe the characters of the system and use a state equation model to estimate the utilization of servers and cycle time of system with the consideration of both single- and dual-command cycle times. Based on this work, Malmborg [6] attempts to propose an optimal system design through comparing AVS/RS systems to traditional autonomous storage systems, and his results shows the former one has advantages in cost-savings and operational flexibility. And Malmborg [7] extends the previous works by considering the opportunistic-interleaving in the system. By using queueing method, the activity of vehicle is modeled by an M/G/V queue and the activity of lifts are modeled by a G/G/L queue in the research of Fukunari and Malmborg [8]; Kuo et al. [9]. Roy et al. [10] analyze a single-tier AVS/RS using semi-open queueing network and examines the system performance. Furthermore, as a useful modelling tool, semi-open queueing network is used to model AVS/RS by the works of Heragu et al. [11] and Marchet et al. [12]. Nevertheless, there are many researches analyze AVS/RS through simulation, such as Ekren [4]. Recently, Ekren and Akpunar [13] develop an open queuing network and a software-based tool to calculate the performance of AVS/RS. In this research, they consider both single- and dual-command cycles, and also estimate system performance related to energy consumption.

For the shuttle-based storage and retrieval systems (SBS/RS), some studies focus on the system design, energy consumption and scheduling process. For instance, Zhao et al. [14] use a semi-open queueing network (SOQN) to model a tier-to-tier SBS/RS system to identify the optimal number of shuttles and provide some insights in system design. Through simulation analysis, Ha and Chae [15] propose a free balancing in SBS/RS systems to prevent collisions and blockages and achieve the targeted system throughput with an optimal number of shuttles. Wu et al. [16] build a queueing model and design an optimal algorithm to find the minimum cost configurations in terms of number of tiers, aisles, lifts and workstations with given throughput, tote capacity and order cycle time requirements. Lei et al. [17] investigate the optimal storage location assignment by using a optimization model. Besides, Luo et al. [18] and Dong et al. [19] investigate the optimal scheduling rule for storage and retrieval processes, respectively, to minimize the makespan of storing or retrieving a series of loads. And Liu et al. [20] develops an energy consumption model for the SBS/RS and estimate the maximum energy consumption under different throughput requirement.

Studies on the shuttle-based compact storage systems are scant, notwithstanding its better volume flexibility, lower operational cost and shorter respond time. Tappia et al. [2] consider multiple tiers and build a semi-open queueing network to model this system. Based on the results of their analytical models, they show the optimal depth/width ratio and number of tiers and compare the economic performance between specialized and generic shuttles. Compared to the research of Tappia et al. [2], Manzini et al. [21] only focus on the estimation of travel time and distance, aiming to find an appropriate layout and system configuration to optimize the system performance in terms of travel distance and cycle time. Borovinšek et al. [3] attempt to find out the optimal layout and system configuration to minimize the investment, energy consumption and cycle time of the system by using a multi-objective optimization model. D’Antonio et al. [22] consider the effect of different allocation criterion on system performance and propose an analytical model based on probabilistic approach to estimate the cycle time and its standard deviation. Boysen et al. [23] focus on a shuttle-based deep-lane storage system with forklifts performing vertical movements. They build a mixed-integer programming model to estimate the performance of two system configurations, namely one-sided and two-sided access to deep-lane storage system, aiming at avoiding blocking. Eder [24] proposes a continuous-time open queueing network taking into account the effect of capacity limitation and the results show that as the increasing of storage depth, the throughput time increases and the investment cost decreases. Recently, Kumawat and Roy [25] develop a new solution approach to solve the multi-stage semi-open queuing networks and apply it in the shuttle-based compact storage systems, which is more accurate for estimating system performance.

Our literature review shows that a sequential processing policy is used to manage the movement of shuttles and transfer car. For instance, when a retrieval transaction is assigned to a shuttle, the shuttle travels to the first bay of its lane and place a request for transfer car sequentially. Once the transfer car is available, it moves to shuttle’s lane, transports the shuttle to the retrieval lane, releases the shuttle, waits for shuttle retrieving the load and then transports the shuttle to the I/O point. During the retrieval transaction, the transfer car cannot respond for demand of any other shuttles, which means the longer the time that the shuttle retrievals load takes, the more inefficient the whole system will be. As pointed out by Tappia et al. [2], the sequential processing policy is currently in use for some warehouses since their storage lanes are not too deep. As the storage lanes become deeper, however, it will take more time for transfer car in waiting for shuttles retrieving loads. Under the parallel processing policy, the movements of shuttles within storage lanes and transfer car in the cross-aisle are simultaneous (Fig 1). Some previous studies have examined the performance of such a policy in automated and vehicle-based storage and retrieval systems [26, 27]. The systems in their studies are crane-based [26] or single/double deep storage systems [27], which are differ from the shuttle-based compact storage systems discussed in our study. Besides, the former research uses deterministic models and the latter only takes retrieval transactions into consideration. Recently, Kumawat et al. [28] propose a closed queueing network with two-phase servers to model the simultaneously operations of shuttle and transfer car in a shuttle-based compact storage system. However, their model only captures the parallel movements of shuttles and transfer car before their joint movement, meaning the transfer car still has to wait for shuttle moving within storage lane to pick up the load.

In summary, simultaneously operations of independent resources in autonomous storage and retrieval systems have attracted the attention of scholars who have performed a number of theoretical studies. However, existing studies on shuttle-based compact storage systems either assume sequential operations between shuttle and transfer car or focus on the modeling of parallel movements of shuttle and transfer car before their joint movement in cross-aisle, both of which mean that the transfer car has to wait for shuttle retrieving the load. In practice, the simultaneous movements of different resources when processing a transaction have advantages in system performance over their sequential movements [28]. Despite the requirement for shorter response times and the performance benefits of parallel processing policy, most previous literatures mainly focus on the sequential processing policy and studies on the parallel processing policy are rare.

Therefore, to contribute to the scant literature on this subject, this study aims to estimate the system performance under parallel processing policy and investigate the conditions on which the parallel processing policy outperforms the sequential processing policy. Based on this, this study analyzes the operational processes of shuttle-based compact storage systems under parallel processing policy and develop a multi-class semi-open queuing network (SOQN) with class switching to model such system. Meanwhile, a fork-join queueing network (FJQN) is used to model the concurrent movement of shuttles and transfer car. Since the original network does not have a product-form solution, a decomposition-based approximation approach is developed to estimate the system performance and simulation is used to validate the accuracy of the analytical model. Additionally, a series of numerical experiments are conducted to compare the system performance under parallel and sequential processing policies. Some design insights and managerial implications are provided through the investigation of a real case. With respect to the previous literature, this study mainly focuses on the parallel movements of shuttles and the transfer car. The results may provide new insights for the improvement of warehouse performance. The main contributions of this study are the followings:

1. We develop a SOQN combined with FJQN to model the parallel movements of shuttles and the transfer car in shuttle-based compact systems. Compared to the previous studies, our model is stochastic and considers both storage and retrieval transactions, thereby taking into account the effect of time spent on waiting for resources to be paired and the route of shuttles. Besides, our model allows the transfer car to be released and respond for the demand of another shuttles when the shuttle is retrieving the load (in the existing studies, the transfer car have to wait for shuttle moving within storage lane to pick up the load).
2. We validate the proposed model using numerical experiments and apply the model on a real case and compare it with the model under sequential processing policy proposed by Tappia et al. [2]. Our analytical results provide some managerial insights in regards to the conditions in terms of number of shuttles, depth/width ratio and arrival rate of orders, under which the parallel processing policy should be considered.

The rest of the study is organized as follows: section 2 provides the system description and assumptions. In section 3, we introduce the models and the approximate solution approach is described in section 4. Section 5 contains the simulation validation, numerical experiments and the insights. Conclusions and future works are presented in section 6.

## 2 System description and assumptions

### 2.1 Main notations and assumptions

Table 1 summarized main notations used throughout the study.

**Table 1:** Main notations.

Notation	Description
*λ*_*r*_, *λ*_*s*_	Arrival rate of retrieval and storage transactions
*N*_*s*_	Number of shuttles
*N*_*c*_, *N*_*l*_	Number of storage columns and lanes at each side of cross-aisle.
*w*, *d*	Unit width and depth per storage position
*t*_*t*_, *t*_*sh*_	Constant time required for transfer car or shuttle to load/unload the shuttle or unit load
*v*_*t*_, *v*_*sh*_	Constant velocity of transfer car and shuttle
*t*_*sh*1_, *t*_*sh*2_, *t*_*sh*3_	Expected travel time related to shuttles
*t*_*t*1_, *t*_*t*2_, *t*_*t*3_	Expected travel time related to transfer car
*p*_*s*_, *p*_*r*_	Probability of storage and retrieval transaction
*p*_*sin*_, *p*_*sio*_	Probability that a transaction is assigned to shuttle dwelling at interior or I/O point
*p*_*cin*_, *p*_*cio*_	Probability that the transfer car dwells at interior or I/O point
*p*_*ss*_, *p*_*sd*_	Probability that the assigned shuttle is or is not present in the lane where the retrieval load is present
*T*_*ir*_, $T_{iu}^{c}$	Mean service time of node *i* for class *r* customer or chain *u* customer
*e*_*ir*_, $e_{iu}^{c}$	Mean number of visits of a class *r* customer or chain *u* customer at node *i*
*p*_*ir*,*js*_	Probability that a class *r* customer at the *i*th node is transferred to class *s* and the *j*th node
*p*_0,*js*_	Probability that a class *s* customer from outside enters the *j*th node
*p*_*js*,0_	Probability that a class *s* customer leaves the system after the service at the *j*th node
*E*[*T*]	Expected throughput time of the system
*U*_*sh*_, *U*_*t*_	Average utilizations of shuttle and transfer car
*L*_*o*_, *L*_*sh*_	Average number of transactions and free shuttles waiting at external queue of system
*L*_*f*_, *L*_*i*_	Mean queue length at fork-join node and at node *i*
*dw*	The depth/width ratio of system

The following assumptions are made in this study:

1. We only consider a single tier. This is based on the following observations. First, the parallel movements of shuttle and transfer car are performed within a single tier. Second, our model can be easily extended to the case of multiple tiers by using the multi-tier linking approach proposed by Tappia et al. [2].
2. We only consider a system with specialized shuttle, since we are interested in whether the simultaneous operations of shuttle and transfer car improve the system performance.
3. The arrival process of both storage and retrieval transactions are assumed to follow a Poisson distribution.
4. The random storage policy is used, meaning the probability of a product being stored in any storage positions is equal.
5. We consider the storage system operates in single-command cycles, which means only a single storage transaction or a single retrieval transaction is performed and only one unit load is handled in each cycle.
6. Each storage lane holds one product.
7. Since compared with the number of storage lanes, the number of shuttles is small, we assume a storage lane can be accessed by at most one shuttle once so that we can ignore the shuttle blocking effects within a storage lane.
8. The shuttles and the transfer car follow a point-of-service-completion (POSC) dwell point policy. Therefore, the shuttles and the transfer car will wait either at an interior point after completion of a storage transaction or the I/O point after completion of a retrieval transaction.
9. The shuttles and the transfer car follow a first-come-first-served (FCFS) scheduling policy.
10. The arriving transaction is performed by the first available shuttle, or by the first shuttle waiting at the idle shuttle queue regardless of the transaction type and shuttle dwell point.
11. We do not consider the effect of acceleration and deceleration on the movement of shuttles and transfer car.

### 2.2 System and operational process description

Fig 1 provides a top view of the studied system. A single tier shuttle-based compact storage system with specialized shuttles consists of multiple storage lanes with each lane holding one product. A cross-aisle is located in the middle of the tier, which is orthogonal to the storage lanes. The movement within the storage lanes is performed by shuttles. In the meantime, a transfer car performs the movement along the cross-aisle. There is only one input/output (I/O) point, which is located at the corner of storage lanes and the end of cross-aisle. Shuttles waiting positions are located next to the I/O point. A conveyor moves the loads to be stored from the inbound work station to the shuttle waiting position and the loads to be retrieved from the shuttle waiting position to the outbound work station.

**Fig 2:**
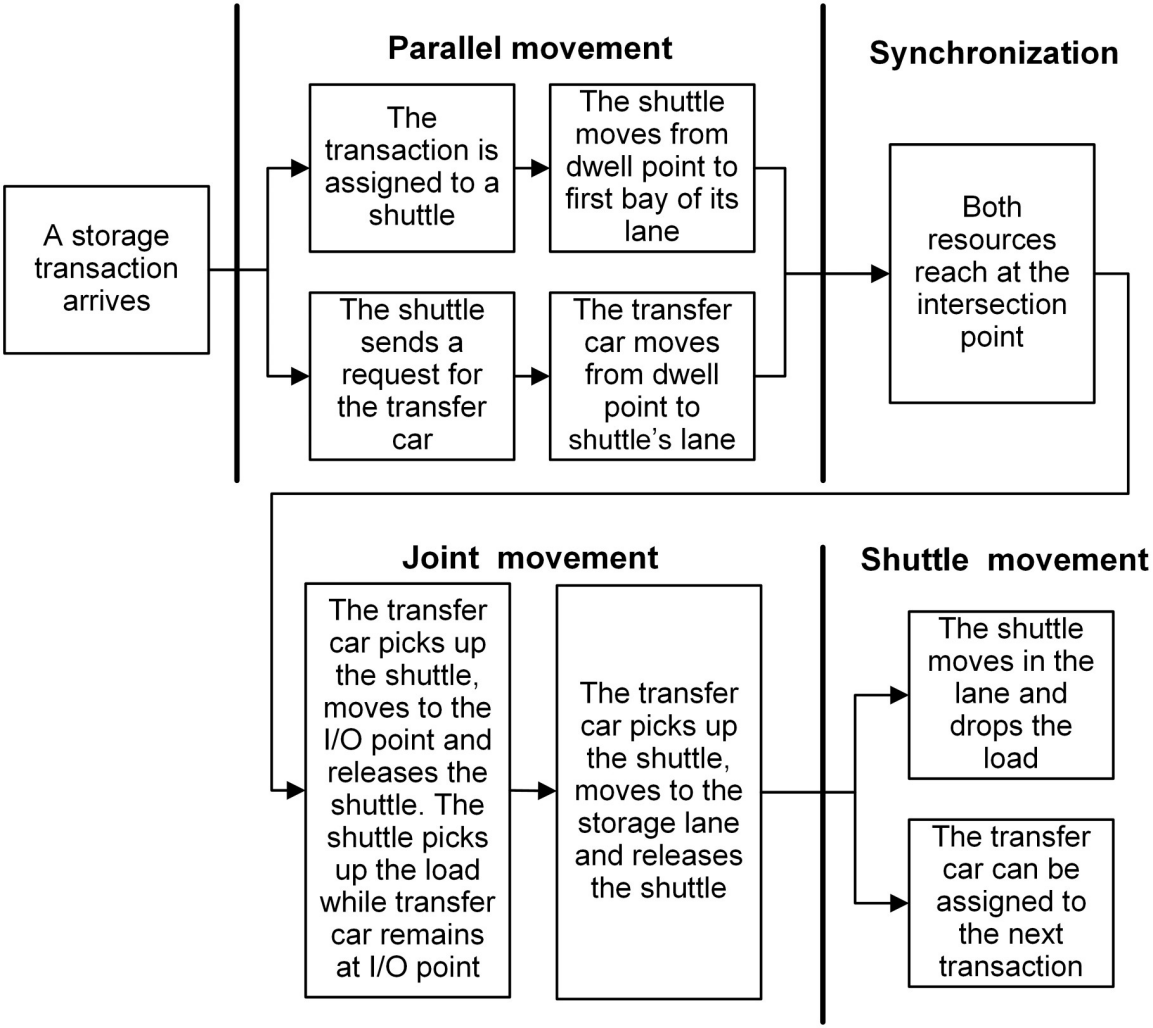
Operational process for storage transactions when the shuttle dwells at an interior point.

**Fig 3:**
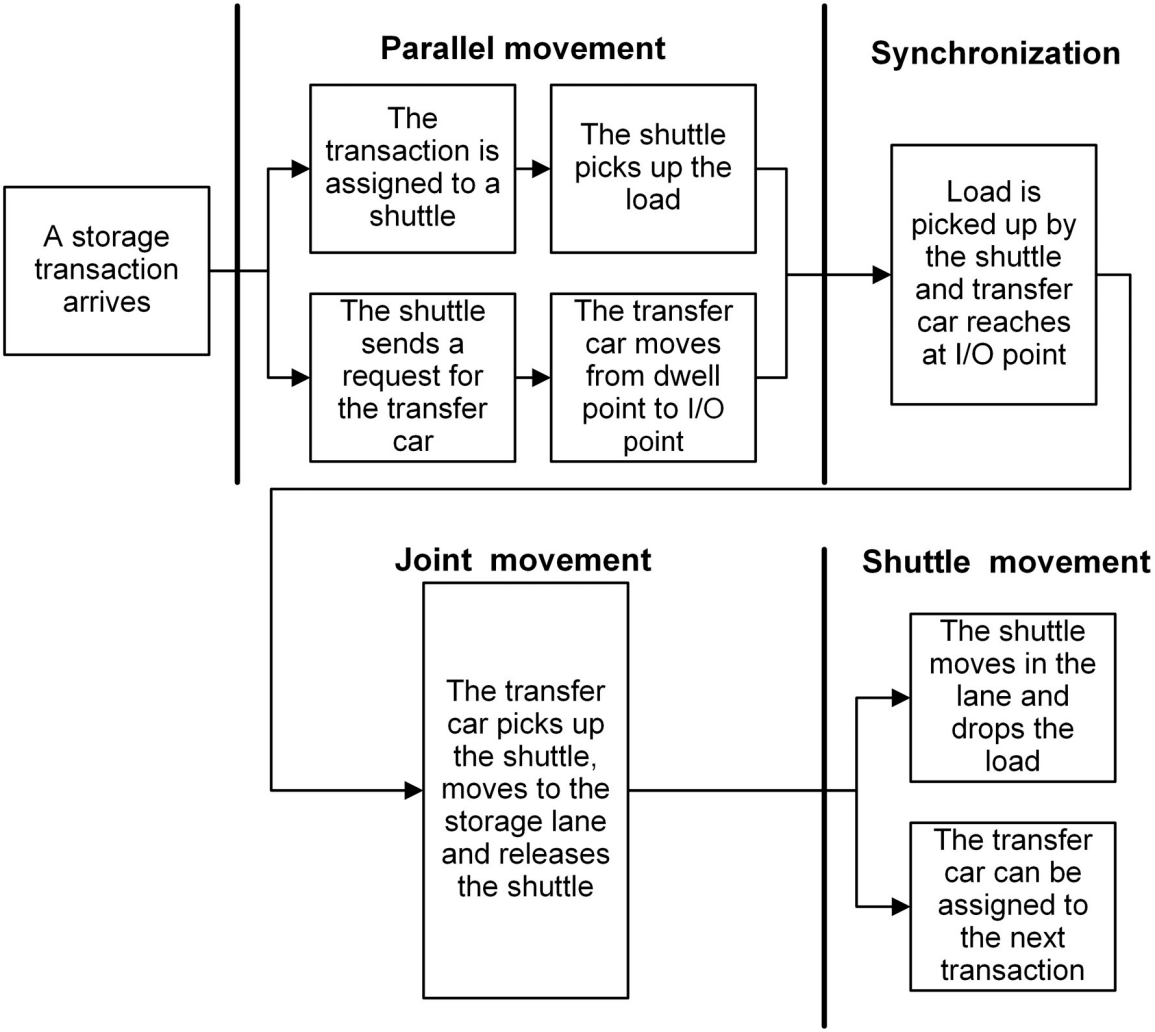
Operational process for storage transactions when the shuttle dwells at the I/O point.

**Fig 4:**
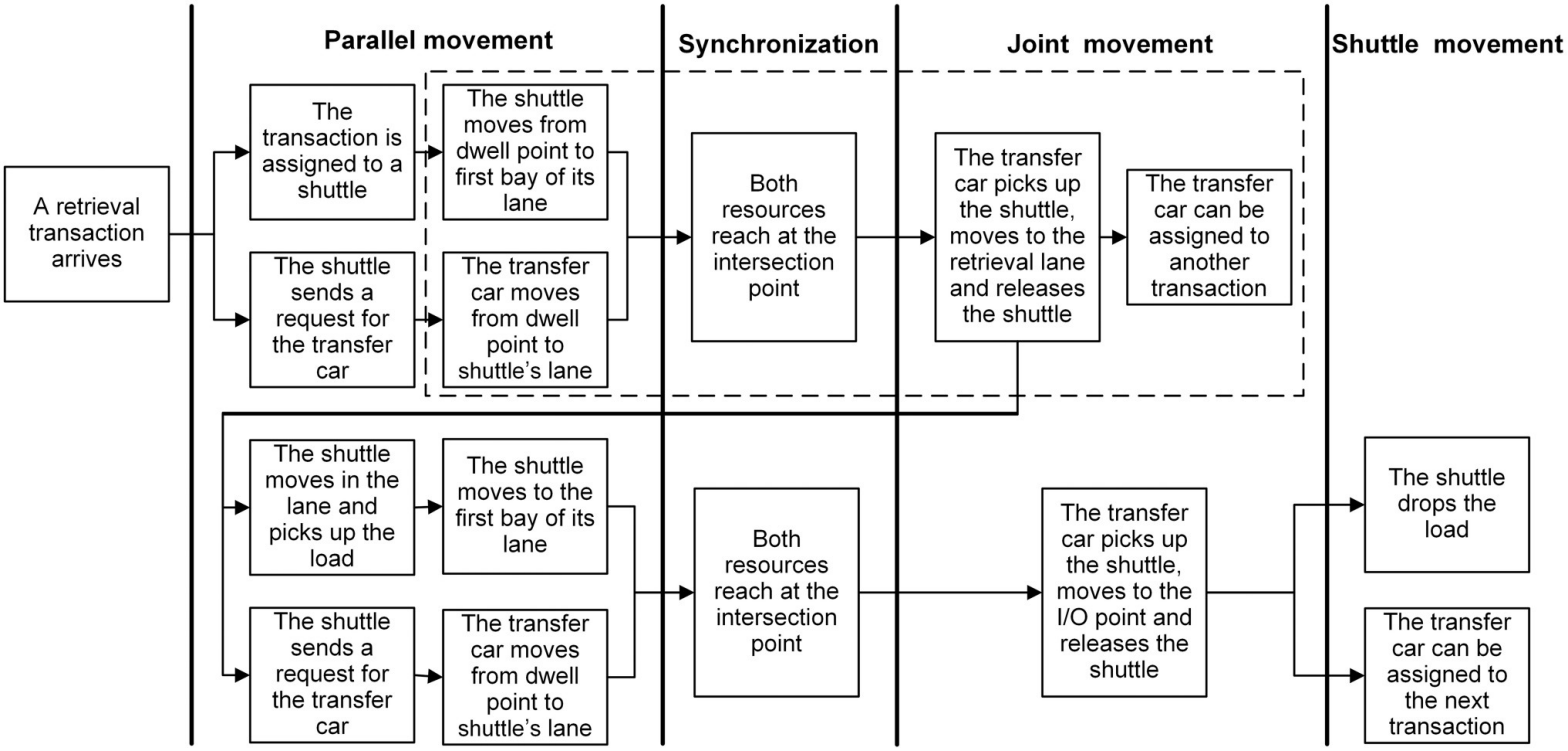
Operational process for retrieval transactions when the shuttle dwells at an interior point. The operational steps within dotted line are performed only when the shuttle is not present in the lane where the retrieval load is present.

**Fig 5:**
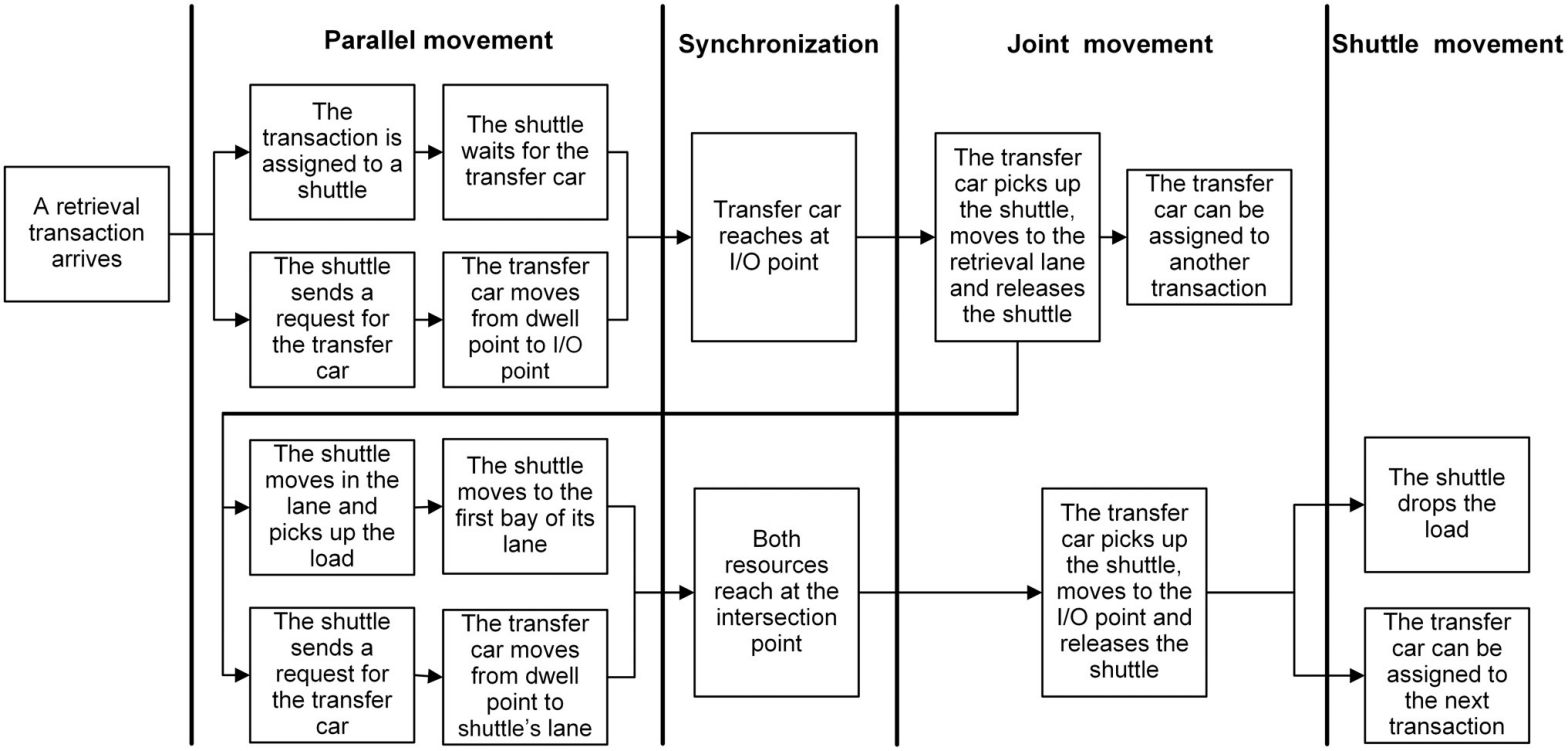
Operational process for retrieval transactions when the shuttle dwells at the I/O point.

When a transaction is assigned to a shuttle, a request is made by the shuttle for transfer car simultaneously. Given the POSC and FCFS policies, the shuttle and transfer car can dwell at any interior or I/O point and a transaction can be assigned to any shuttle regardless of its dwell point. Besides, whether the shuttle dwells at the same lane of retrieval position results in different individual movements required to perform retrieval transactions. Therefore, depending on the dwell point of shuttle (interior or I/O point) and the type of transaction (storage or retrieval), one of the following scenarios showed in Figs 2–5 can occur (For details about the operational processes of such a system under sequential processing policy, we refer to Tappia et al. [2]).

### 2.3 Components of travel time related to shuttles and transfer car

Given the random storage policy and the operational processes showed in Figs 2–5, the expected travel time related to shuttles and transfer car be obtained based on the probability distribution of storing or retrieving a load from each storage position, i.e., a uniform distribution. Therefore, each component of the travel time related to shuttles and transfer car can be expressed as follows:

Time required for the shuttle to:

1. travel from dwell point (or the retrieval position) to the first bay of its lane:
$$t_{sh1}=\sum_{k=1}^{N_{c}}\frac{1}{N_{c}}\frac{\left(k-1\right)d}{v_{sh}}=\frac{\left(N_{c}-1\right)d}{2v_{sh}}$$ (1)
2. travel from dwell point to the retrieval position when dwells in the same lane of retrieval position:
$$t_{sh2}=\sum_{i=1}^{N_{c}}\sum_{j=1}^{N_{c}}\frac{1}{N_{c}^{2}}\frac{\left|i-j\right|d}{v_{sh}}$$ (2)
3. pick up or drop the load:
$$t_{sh3}=t_{sh}$$ (3)

Time required for the transfer car to:

1. travel from I/O point to shuttle’s lane or travel from its dwell point (not I/O point) to I/O point:
$$t_{t1}=\frac{N_{l}w}{2v_{c}}$$ (4)
2. travel from dwell point (not I/O point) to the shuttle’s lane:
$$t_{t2}=\sum_{i=1}^{N_{l}}\sum_{j=1}^{N_{l}}\frac{1}{N_{l}^{2}}\frac{\left|i-j\right|w}{v_{c}}$$ (5)
3. load or unload the shuttle:
$$t_{t3}=t_{t}$$ (6)

## 3 Semi-open queueing network for shuttle-based compact storage systems

### 3.1 Queueing model

**Fig 6:**
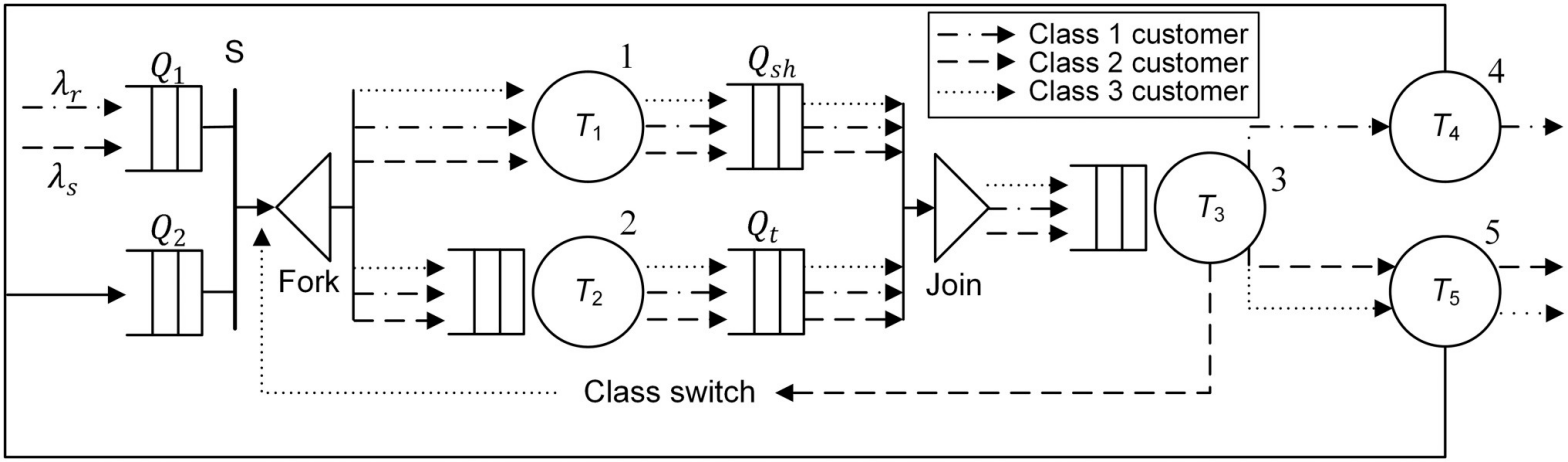
Queueing network model of the shuttle-based compact storage system.

Fig 6 shows the SOQN model for the shuttle-based compact storage systems. For modeling purpose, we divide the system operational process into three parts: parallel movement, joint movement and shuttle movement (Figs 2–5). The model considers both storage and retrieval transactions. The shuttles are modeled as resources.

As showed in Fig 6, there are three nodes and a fork-join network with two nodes. All the service required for shuttles and transfer car to complete the parallel movement is modeled by the fork-join network, in which the service of shuttles is represented by an infinite-server (IS) node 1 and the service of transfer car is represented by a single-server node 2. The joint movement is captured by the single-server node 3. IS nodes 4 and 5 represent shuttle movements for retrieval and storage transaction, respectively. Node S is a synchronization station with two queues that *Q*_1_ represents the external queue of transactions and *Q*_2_ represents the queue where shuttles will be released to after completing service. Under the parallel processing policy, an incoming transaction, after being paired with the first available shuttle, is split into two parts that one is served by the shuttle and the other by the transfer car. The completed part waits in one of the two join queues denoted by *Q*_*sh*_ (represents the shuttles) and *Q*_*t*_ (represents the transfer car) for the completion of the other one. Then they join at the join node.

Our model allows transfer car performing other tasks after it releases the shuttle to retrieve the required load. This results that the shuttle and transfer car have to be synchronized upon completion of their parallel movements more than once when processing retrieval transactions (Figs 4 and 5). To deal with the differences between their first and second synchronizations, class switching is allowed in our model. Specifically, we call the transaction the customer of the system, and storage and retrieval transaction as a class 1 and 2 customer, respectively. In the case that a class 2 customer is served by a shuttle dwelling in a storage lane which is different from that of retrieval position, after the transfer car moving to the destination storage lane and unloading the shuttle (the first joint movement which is captured by node 3), the class of the customer changes to 3. While in the case that a class 2 customer is served by a shuttle dwelling at I/O point, after the transfer car moving from I/O point to destination lane and unloading the shuttle, the class of the customer also changes to 3. Table 2 describes the customer class switching rule.

**Table 2:** Description of the customer class switching rule.

Customer class prior to the joint movement	Dwell point of shuttle	Same lane	Customer class after the joint movement
1	I/O or interior point		1
2	Interior point	yes	2
2	Interior point	no	3
2	I/O point		3
3			3

Same lane means whether the shuttle is present in the lane where the retrieval load is present.

The external arrival process of transactions is assumed to follow a Poisson distribution. Thus, the type of transaction waiting at the head of *Q*_1_ can be storage with probability *p*_*s*_ = *λ*_*s*_⁄(*λ*_*r*_ + *λ*_*s*_) or retrieval with probability *p*_*r*_ = *λ*_*r*_⁄(*λ*_*r*_ + *λ*_*s*_). The probability with which a transaction is assigned to shuttle dwelling at interior (or I/O) point is *p*_*sio*_ = *λ*_*r*_⁄(*λ*_*r*_ + *λ*_*s*_) (or *p*_*sin*_ = *λ*_*s*_⁄(*λ*_*r*_ + *λ*_*s*_)). Since the loads are stored randomly in the system, we can get *p*_*ss*_ = 1⁄(2*N*_*l*_) and *p*_*sd*_ = (2*N*_*l*_ − 1)⁄(2*N*_*l*_).

Transfer car dwells at interior or I/O point depends upon the previous task it completes (there are three possible tasks in our model: joint movement of storage transaction and the first and second joint movement of retrieval transaction), which makes it difficult to calculate the corresponding probabilities. Specifically, the transfer car dwells at I/O point after the completion of the first joint movement of retrieval transaction, while it dwells at an interior point after the completion of the joint movement of storage transaction and the second joint movement of retrieval transaction. Given the random storage policy, it is reasonable to assume that, in steady state, the number of class 2 customers (excluding the case that shuttle is present in the lane where the retrieval load is present) is equal to that of class 3 customers. This assumption implies, given that the shuttle is not present in the lane where the retrieval load is present, the probability that the transfer car performs the first joint movement of retrieval transaction is equal to the probability that it performs the second joint movement of retrieval transaction (i.e., both probabilities can be expressed as (*p*_*r*_*p*_*sin*_*p*_*sd*_ + *p*_*r*_*p*_*sio*_)/2). Thus, we can obtain:

$$p_{cin}=\frac{1}{2}\left(p_{r}p_{sin}p_{sd}+p_{r}p_{sio}\right)+p_{s}=\frac{\left(2\lambda_{r}^{2}+6\lambda_{r}\lambda_{s}+4\lambda_{s}^{2}\right)N_{l}-\lambda_{r}\lambda_{s}}{4{\left(\lambda_{r}+\lambda_{s}\right)}^{2}N_{l}}$$ (7)

$$p_{cio}=\frac{1}{2}\left(p_{r}p_{sin}p_{sd}+p_{r}p_{sio}\right)+p_{r}p_{sin}p_{ss}=\frac{\left(2\lambda_{r}^{2}+2\lambda_{r}\lambda_{s}\right)N_{l}+\lambda_{r}\lambda_{s}}{4{\left(\lambda_{r}+\lambda_{s}\right)}^{2}N_{l}}$$ (8)

Let *f* denotes the fork-join node, the routing probabilities are given as follows:

$$\begin{array}{l} p_{0,f1}=p_{s},p_{f1,31}=1,p_{31,41}=1,p_{41,0}=1\\ p_{0,f2}=p_{r},p_{f2,32}=1,p_{32,52}=p_{sin}p_{ss},p_{32,f3}=p_{sin}p_{sd}+p_{sio},p_{52,0}=1\\ p_{f3,33}=1,p_{33,53}=1,p_{53,0}=1 \end{array}$$

### 3.2 Service time expressions

The service time of each node for each class customer depends upon the type of transactions and the dwell point of shuttles and transfer car. Therefore, based on the scenarios provided in Figs 2–5 and the component of travel times related to shuttles and the transfer car, we calculate the service time expressions for nodes 3, 4 and 5 and summarize in Table 3 and fork-join node in Table 4, as well as their corresponding probabilities and scenarios.

**Table 3:** Service time expressions for nodes 3, 4 and 5.

Node	Notation	Mean	Node	Notation	Mean	Probability	Corresponding scenario
4	*T*_4_	*t*_*sh*1_ + *t*_*sh*3_	3	*T*_31_	2*t*_*t*1_ + 2*t*_*t*2_ + *t*_*t*3_	*p*_*sin*_	Fig 2
					*t*_*t*1_ + 2*t*_*t*3_	*p*_*sio*_	Fig 3
				*T*_32_	*t*_*t*2_ + 2*t*_*t*3_	*p*_*sin*_ *p*_*sd*_	Fig 4, different lane
5	*T*_5_	*t*_*sh*3_			*t*_*t*1_ + 2*t*_*t*3_	*p*_*sin*_ *p*_*ss*_	Fig 4, same lane
					*t*_*t*1_ + 2*t*_*t*3_	*p*_*sio*_	Fig 5
				*T*_33_	*t*_*t*1_ + 2*t*_*t*3_	1	Fig 5 or different lane in Fig 4

Same or different lane means the shuttle is or is not present in the lane where the retrieval load is present.

**Table 4:** Service time expressions for fork-join node.

Scenario	Customer class	Dwell point		Same lane	Probability	Mean service time	
		Shuttle	Transfer car			Node 1	Node 2
1	1	Interior	I/O		*p*_*s*_ *p*_*sin*_ *p*_*cio*_	*t*_*sh*1_	*t*_*t*1_
2	1	Interior	Interior		*p*_*s*_ *p*_*sin*_ *p*_*cin*_	*t*_*sh*1_	*t*_*t*2_
3	1	I/O	I/O		*p*_*s*_ *p*_*sio*_ *p*_*cio*_	*t*_*sh*3_	0
4	1	I/O	Interior		*p*_*s*_ *p*_*sio*_ *p*_*cin*_	*t*_*sh*3_	*t*_*t*1_
5	2	Interior	I/O	yes	*p*_*r*_ *p*_*sin*_ *p*_*cio*_ *p*_*ss*_	*t*_*sh*1_ + *t*_*sh*2_ + *t*_*sh*3_	*t*_*t*1_
6	2	Interior	Interior	yes	*p*_*r*_ *p*_*sin*_ *p*_*cin*_ *p*_*ss*_	*t*_*sh*1_ + *t*_*sh*2_ + *t*_*sh*3_	*t*_*t*2_
7	2	Interior	I/O	no	0.5*p*_*r*_*p*_*sin*_*p*_*cio*_*p*_*sd*_	*t*_*sh*1_	*t*_*t*1_
8	2	Interior	Interior	no	0.5*p*_*r*_*p*_*sin*_*p*_*cin*_*p*_*sd*_	*t*_*sh*1_	*t*_*t*2_
9	2	I/O	I/O		0.5*p*_*r*_*p*_*sio*_*p*_*cio*_	0	0
10	2	I/O	Interior		0.5*p*_*r*_*p*_*sio*_*p*_*cin*_	0	*t*_*t*1_
11	3		I/O		0.5*p*_*r*_*p*_*cio*_	2*t*_*sh*1_ + *t*_*sh*3_	*t*_*t*1_
12	3		Interior		0.5*p*_*r*_*p*_*cin*_	2*t*_*sh*1_ + *t*_*sh*3_	*t*_*t*2_

## 4 Solution approach for semi-open queueing networks

**Fig 7:**
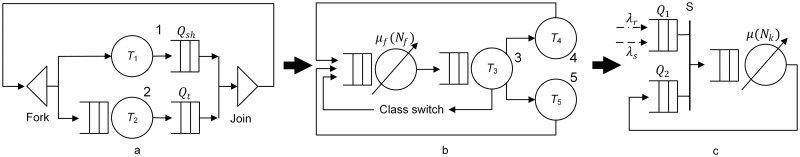
Procedure for reducing the original network.

The queueing model we developed is a multiclass semi-open queueing network with both general and infinite stations. It is difficult to evaluate such queueing network directly by continuous-time Markov chain (CTMC) since the system has a large state space. This is because we have to record the number of each customer class in each node and its corresponding queue, the number of idle shuttles and the exact order of all of the customers in join queues. In order to estimate the performance of such model with a non-product form solution, we develop a decomposition-based approximation method including following three steps: first, we consider the FJQN as a closed network and estimate its load-dependent service rate; second, we replace the FJQN by a Flow Equivalent Server (FES), aggregate the compliment network, together with the FES, into a single server and calculate its service rate; at last, we solve the reduced SOQN with one single server directly by CTMC. Fig 7 shows the procedure for reducing the original network to a one single-server network.

### 4.1 Estimation of load-dependent service rate of the FJQN

There are two single-server stations in the FJQN, one of which is general station representing transfer car and the other is IS representing shuttles (Fig 7a). Note that there is no class switching in this closed queueing network. Thus, to obtain the service rate of FJQN, we first aggregate all classes into one, as suggested by [29], and consider the FJQN as a closed network and short-circuit the other nodes. Thus, we can approximate the state probabilities and calculate the service rate.

The mean service time of node 1 and 2 for the aggregation class is given by the combination of mean service time of all possible scenarios. Therefore, the mean service time of node1 and 2, as well as their second moments are obtained by:

$$E(T_{i})=\sum_{m}p_{m}T_{im}$$ (9)

$$E(T_{i}^{2})=\sum_{m}p_{m}T_{im}^{2}$$ (10)

where *E*(*T*_*i*_) denotes the mean service time of node *i*, *T*_*im*_ represents the mean service time of node *i* in *m*th scenario described in Table 3 with its corresponding probability denoted by *p*_*m*_ and *E*(*T*_*i*_^*2*^) represents the second moment of expected service time for the aggregation class. Moreover, the squared coefficient of variation (scv) of the service time can be obtained by $cv_{i}^{2}=\left(E(T_{i}^{2})-E{\left(T_{i}\right)}^{2}\right)/E{\left(T_{i}\right)}^{2}$.

Since the service time of two nodes are general distributed and $cv_{i}^{2}<1$, an Erlang-*k* distribution is adopted to approximate the service process of each node, where *k* denotes the number of exponential phases and *k* = [1/*cv*^2^]. The mean service time at each phase, *μ*^−1^ = *E*(*T*)⁄*k*.

When a transaction goes through the FJQN, it will be split into two parts, one of which requests the service of shuttles at node 1, the other requests the service of transfer car at node 2. The joining of these two tasks at the join node of FJQN represents the service completion of a transaction. Thus, the state of the system can be described by a two-dimensional vector *st*_*q*_ = (*N*_*ws*_, *N*_*wt*_), where *N*_*ws*_ is the number of waiting shuttles in the join queue *Q*_*sh*_ and *N*_*wt*_ is the number of waiting transfer cars in the join queue *Q*_*t*_. Let *N*_*f*_ be the number of customers in the closed network, *q* be the total number of states in this network, we can obtain *q* = *N*_*ws*_(*N*_*f*_ + 1) + *N*_*wt*_. Given the fact that there is only one transfer car in the system, the state space can be expressed by:

$$N_{ws}+N_{wt}<N_{f}+1,\;N_{ws}=0,1,\cdots N_{f},\;N_{wt}=0,1$$ (11)

As shown in [27], the joining of two tasks can be completed when a shuttle finishes its service if *N*_*wt*_ = 1 or the transfer car finishes its service if *N*_*ws*_ > 1. Thus, the service rate of FJQN can be calculated by:

$$\mu_{f}\left(N_{f}\right)=\sum_{N_{ws}=1}^{N_{f}}\frac{\pi \left(N_{ws},0\right)}{E(T_{2})}+\sum_{N_{ws}=0}^{N_{f}-1}\frac{\pi \left(N_{ws},1\right)}{E(T_{1})}$$ (12)

where *π*(*st*_*q*_) denotes the state probability of state *st*_*q*_. Obviously, the state probabilities *π*(*st*_*q*_) can be obtained by solving the following:

$$\left\{\begin{array}{l} \pi Q=0\\ \pi e=1 \end{array}\right.$$ (13)

where ***e*** is the column vector of ones, ***Q*** denotes the transition matrix of *st*_*q*_ (S1 File).

### 4.2 Solution to the closed queueing network

After obtaining the service rate of FJQN, we replace the FJQN by a FES node with exponential distributed load-dependent service time, *μ*_*f*_(*N*_*f*_). Then the network made up by all nodes (except for the synchronization node), are considered as a closed network (Fig 7b). Since class switching is allowed in this closed queueing network, the switch from classes to chains is needed [30]. According to the concept of chains, there are two chains in the closed queueing network denoted by *c*_1_ and *c*_2_. Without loss of generality, we let *c*_1_ = {1} represents storage transactions, *c*_2_ = {2,3} represents retrieval transactions and *N*_*k*_ denotes the number of customers in the closed network. The size of the state space therefore is reduced to:

$$\left(\begin{array}{c} 4\cdot \left|c_{1}\right|+N_{k1}-1\\ 4\cdot \left|c_{1}\right|-1 \end{array}\right)\cdot \left(\begin{array}{c} 4\cdot \left|c_{2}\right|+N_{k2}-1\\ 4\cdot \left|c_{2}\right|-1 \end{array}\right)$$

where |*c*_*u*_| and *N*_*ku*_ denote the number of elements and customers in chain *c*_*u*_, *u* = 1,2, respectively.

The routing probabilities are given by:

$$\begin{array}{l} p_{f1,31}=1,p_{31,41}=1,p_{41,f1}=1\\ p_{f2,32}=1,p_{32,52}=p_{sin}p_{ss},p_{32,f3}=p_{sin}p_{sd}+p_{sio},p_{52,f2}=1\\ p_{f3,33}=1,p_{33,53}=1,p_{53,f2}=1 \end{array}$$

The visit ratios in a chain are given by:

$$e_{ir}=\sum e_{js}p_{js,ir}\;for\;\left\{\begin{array}{c} r,s\in c_{u}\\ i,j=f,3,4,5\\ u=1,2 \end{array}\right.$$ (14)

After solving the system of linear Eq (14), we can get the visit ratios of the chain to and expected service time at node *i*:

$$e_{iu}^{c}=\frac{\sum_{r\in c_{u}}e_{ir}}{\sum_{r\in c_{u}}e_{fr}}$$ (15)

$$T_{iu}^{c}=\sum_{r\in c_{u}}T_{ir}\cdot \frac{e_{ir}}{\sum_{s\in c_{u}}e_{is}}$$ (16)

Then the throughputs of the closed queueing network, *μ*_1_(*N*_*k*_) for *c*_1_ and *μ*_2_(*N*_*k*_) for *c*_2_, are obtained through mean value analysis (MVA).

### 4.3 Steady state model of reduced SOQN

After substituting the subnetwork made up of all nodes with a FES node (Fig 7c), we first reduce the network into a single chain and then use a birth-death process to model the system [31]. Let the aggregate arrival rate *λ* = *λ*_*s*_ + *λ*_*r*_ be the birth rate and the service rate for the aggregate chain *μ*(*N*_*k*_) = *p*_*s*_*μ*_1_(*N*_*k*_) + *p*_*r*_*μ*_2_(*N*_*k*_) be the load-dependent death rate of the system. The state space is described using a single variable *x*, which represents the number of transactions waiting in queue *Q*_1_ when *x* > 0 and the number of idle shuttles waiting in queue *Q*_2_ when −*N*_*s*_ ≤ *x* ≤ 0. Thus, the load-dependent death rate *μ*(*N*_*k*_) = *μ*(*N*_*s*_ + *x*) when −*N*_*s*_ ≤ *x* ≤ 0 and *μ*(*N*_*k*_) = *μ*(*N*_*s*_) when *x* > 0. The steady state probabilities can be obtained by using flow rate balance equations and can be expressed by (S2 File):

$$\pi \left(x\right)=\left\{\begin{array}{l} \pi \left(-N_{s}\right)\prod_{i=-N_{s}+1}^{x}\frac{\lambda }{\mu \left(N_{s}+i\right)},\;-N_{s}<x\leq 0\\ \pi \left(-N_{s}\right)\prod_{i=-N_{s}+1}^{0}\frac{\lambda }{\mu \left(N_{s}+i\right)}\cdot {\left(\frac{\lambda }{\mu \left(N_{s}\right)}\right)}^{x},\;x>0 \end{array}\right.$$ (17)

$$\pi \left(-N_{s}\right)=\frac{1}{1+\sum_{i=1}^{N_{s}-1}\prod_{k=1}^{i}\frac{\lambda }{\mu \left(k\right)}+\left(\frac{\mu \left(N_{s}\right)}{\mu \left(N_{s}\right)-\lambda }\right)\prod_{k=1}^{N_{s}}\frac{\lambda }{\mu \left(k\right)}}$$ (18)

### 4.4 Performance measures of the system

The expected throughput time of the system, average utilizations of shuttles and transfer car, average queue length of *Q*_1_ are the main performance measures we are interested in and are obtained using the following equations:

$$U_{sh}=\frac{N_{s}-\sum_{i=-N_{s}}^{-1}\left(-i\right)\pi \left(i\right)}{N_{s}}$$ (19)

$$U_{t}=\sum_{x_{t}\in X}p\left(x_{t}\right)$$ (20)

$$L_{o}=\pi \left(0\right)\cdot \frac{\lambda /\mu \left(N_{s}\right)}{{\left[1-\lambda /\mu \left(N_{s}\right)\right]}^{2}}$$ (21)

$$E\left[T\right]=\frac{\sum_{k=1}^{\infty }k\pi \left(k\right)+N_{s}-\sum_{i=-N_{s}}^{-1}\left(-i\right)\pi \left(i\right)}{\lambda }$$ (22)

Where *p*(*x*_*t*_) denotes the probability corresponding to the generic state *x*_*t*_ belonging to ***X***, the set of all possible states *x* of the system, *x*_*t*_ represents the states with the average number of transactions at FJQN, *L*_*f*_ > 0, and node 3, *L*_3_ > 0.

## 5 Model validation and numerical experiments

### 5.1 Model validation

The simulation model is based on Arena software 14.0. Table 5 provides the details about the scenarios in simulation model. The data we use in the validation are derived from the study of Tappia et al. [2] and are provided in Table 6. The depth and width of a tier are measured by the maximum travel distance in the *x*- and *y*-direction, respectively. To validate the analytical model under different resource utilizations, the arrival rate of transactions is set at three levels: 22, 25 and 28 per hour with the assumption that *λ*_*r*_ = *λ*_*s*_, which results a bottleneck utilization ranging from 70% to 90%. 12 scenarios are designed based on the variation of shuttle number and order arrival rate for each combination of depth / width ratio and total number of storage positions. Other assumptions are the same as the analytical model (i.e., POSC dwell point and random storage policy) (S3 File).

**Table 5:** Scenarios generated for model validation.

Depth / Width ratio	Total number of storage positions	Number of shuttles	Arrival rate of transactions	Number of scenarios
1:1	5000	2,3,4,5	22,25,28	12
1:1	10000	2,3,4,5	22,25,28	12
2:1	5000	2,3,4,5	22,25,28	12
2:1	10000	2,3,4,5	22,25,28	12

**Table 6:** Data used for model validation.

Variable	Description	Value
*w*	Unit width per storage position	0.9m
*d*	Unit depth per storage position	1.2m
*t*_*t*_, *t*_*sh*_	Constant time required for transfer car or shuttle to load/unload the shuttle or unit load	5s
*v*_*t*_, *v*_*sh*_	Constant velocity of transfer car and shuttle	1m/s

For each scenario, a warm-up period of more than 5000 transactions is run, followed by 15 replications with a run time of more than 30000 transactions, which leads to a 95% confidence interval where the half-width is less than 2% of the average. Four performance measures are estimated to validate the analytical model: the throughput time of system, the utilizations of transfer car and shuttles and the queue length of *Q*_1_. The accuracy of analytical model is measured by absolute relative error, *ε*, which is defined as *ε* = |*A* − *S*|⁄*S* × 100%, where *A* and *S* denote the analytical and simulation results respectively. The computational complexity of the proposed model can be characterized by *O* (*N*_*s*_^*4*^· max (*N*_*l*_, *N*_*c*_)^2^). In our experiments, the conduction of proposed algorithm takes less than 1 second of computational time on a standard computer.

**Fig 8:**
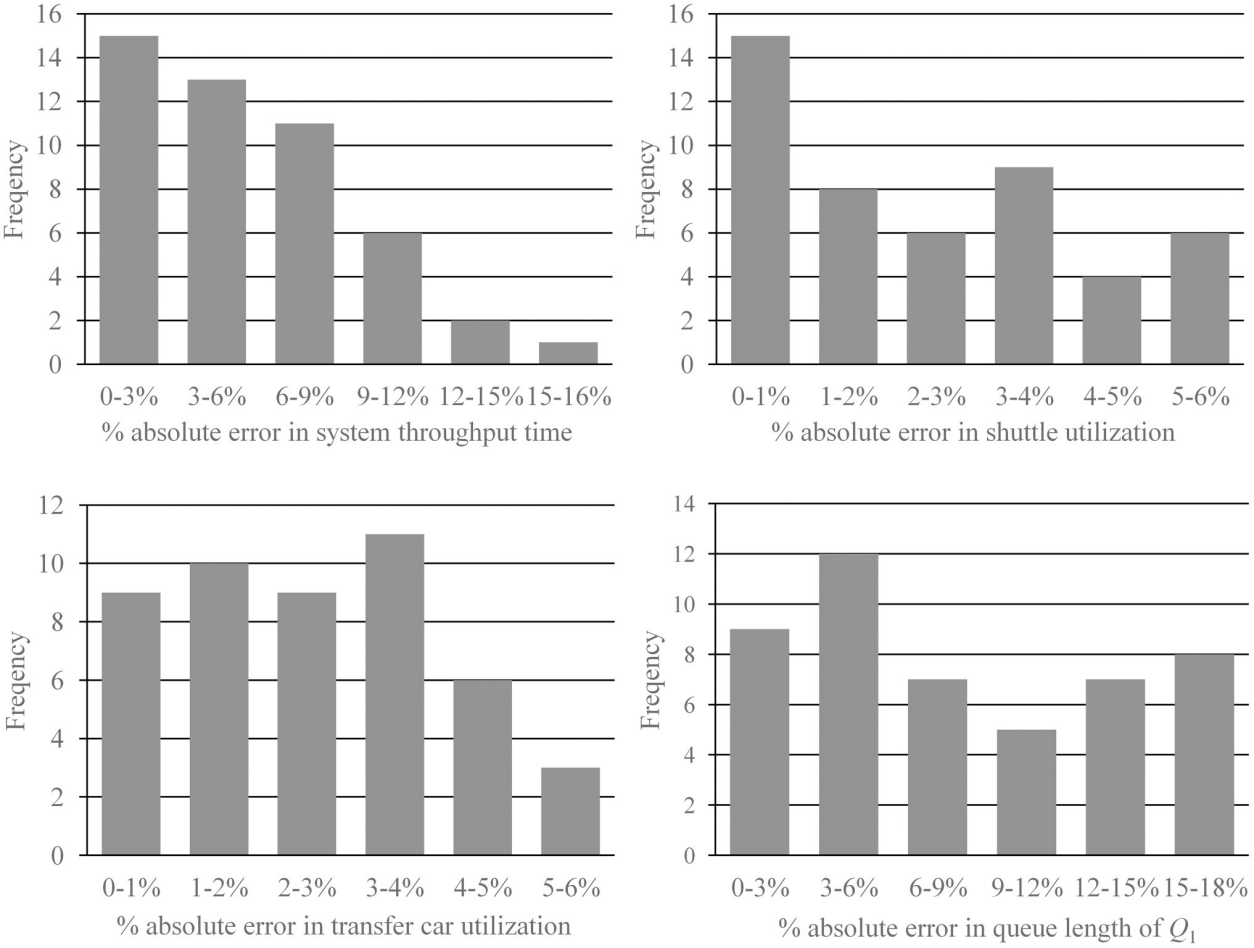
Distribution of absolute errors for performance measures.

The distribution of absolute relative errors for each performance measure is shown in Fig 8. The average absolute errors are 6.32%, 2.93%, 2.38% and 10.81% for expected throughput time, transfer car utilization, shuttle utilization and expected queue length of *Q*_1_, respectively. These results suggest that the analytical model can accurately estimate the system performance.

### 5.2 Comparison of sequential and parallel processing policies

**Fig 9:**
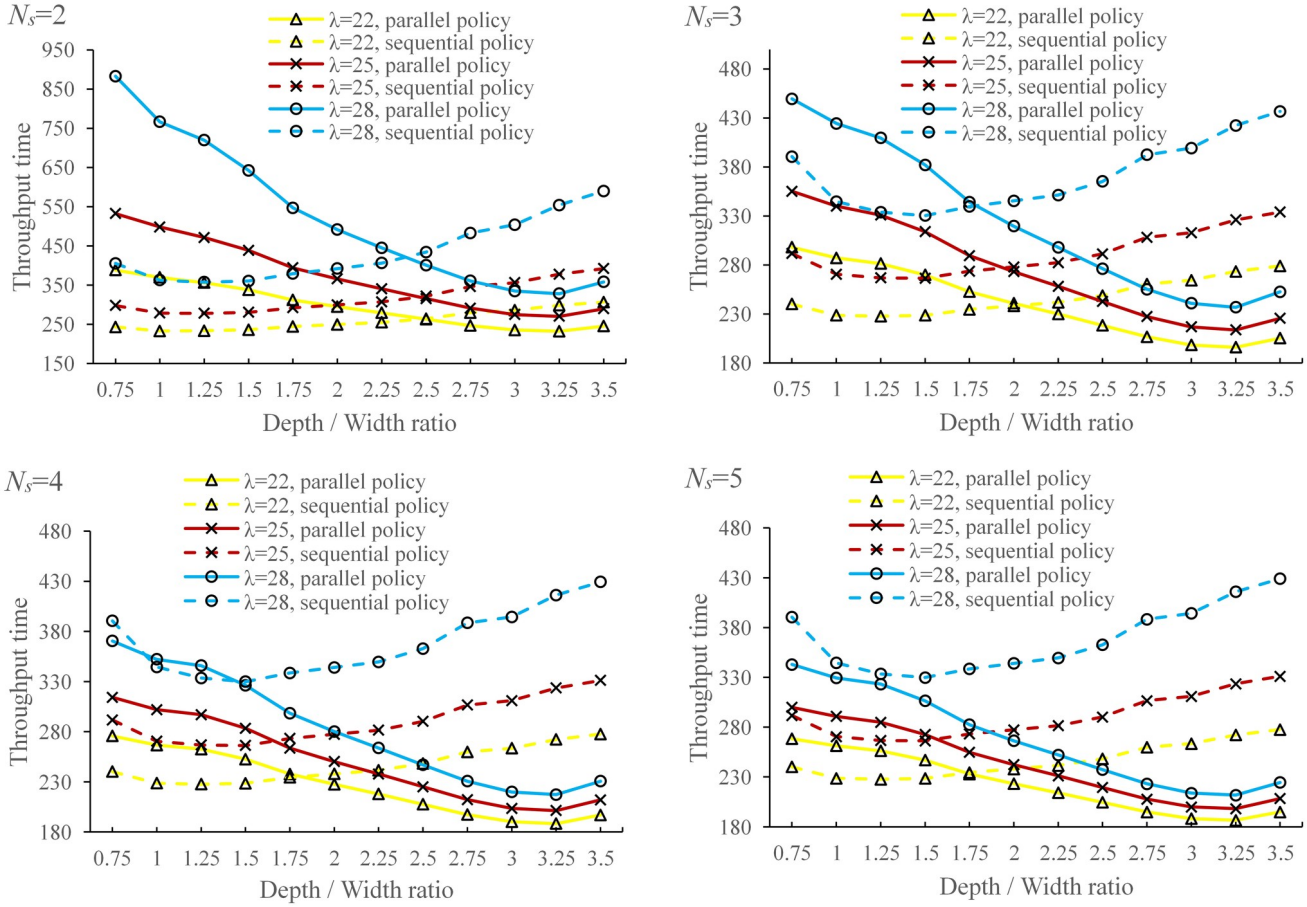
Comparison of parallel and sequential processing policies.

As pointed out by Tappia et al. [2], it may be advantageous for deep lane shuttle-based compact storage systems under parallel processing policy, which means the response time of systems under parallel processing policy may be shorter than that of sequential processing policy with the increase of depth / width ratio. Thus, we compared the performance of two processing policies by carrying out numerical experiments. The system performance under sequential processing policy is estimated using simulation model, while the system performance under parallel processing policy is estimated using analytical model proposed in this study. To compare these two processing polices in more detail, we vary *N*_*s*_ and *λ*, i.e., *N*_*s*_ ranges from 2 to 5 and *λ* varies from18 to 30 with a step size of 0.1 to deal with the uncertainty of order arrival rate and we also assume that *λ*_*r*_ = *λ*_*s*_. The total number of storage positions is 5000. The depth / width ratio varies from 0.75 to 3.5 with a step size of 0.25. The results are shown in Fig 9.

**Fig 10:**
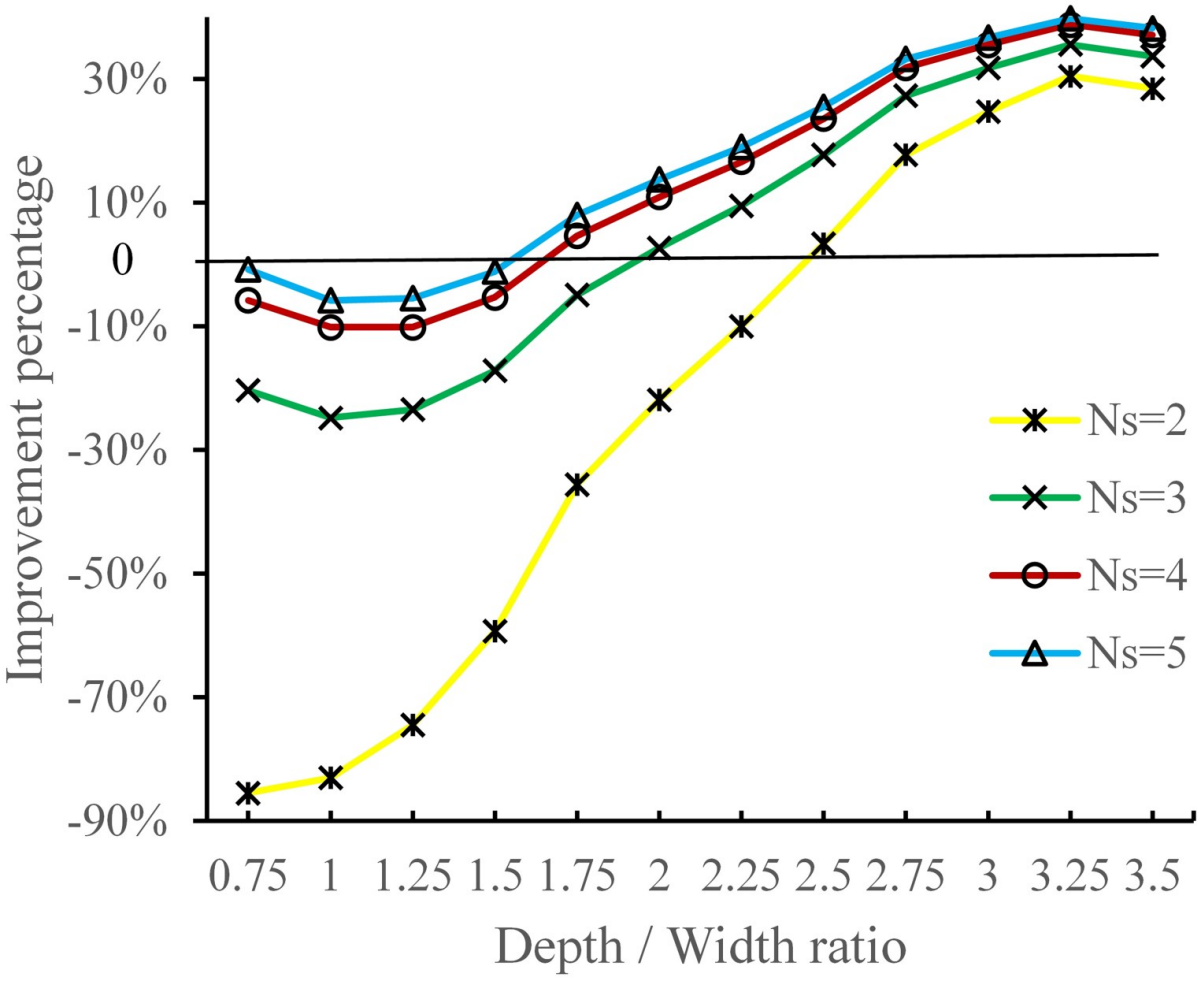
Average improvement percentage of the parallel processing policy over sequential processing policy.

To better understand the difference in system throughput time under different processing policies, we use the average improvement percentage of the parallel processing policy over the sequential processing policy, *I*_*P*_, which is defined by:

$$I_{P}=\frac{1}{V}\sum_{\lambda }\frac{E\left[T_{S}\right]-E\left[T_{P}\right]}{E\left[T_{S}\right]}\times 100\text{\%}$$ (23)

where *E*[*T*_*P*_] and *E*[*T*_*S*_] represent the expected system throughput time under parallel and sequential processing policy, respectively. And *V* is the number of values taken by *λ*. The results are shown in Fig 10.

Obviously, as shown in Fig 7, the average improvement percentage *I*_*P*_ increases with the depth / width ratio and the number of shuttles and the parallel policy performs better than sequential policy when depth / width ratio is large enough. Specifically, there is an intersection point between the curves of two processing policies, denoted by (dw*, *E*[*T*]*). When *N*_*s*_ = 2, *λ* = 28, for example, dw* ≈ 2.37. When *dw* > dw*, the parallel policy outperforms sequential policy. Additionally, dw* decreases with the increase of *N*_*s*_ and *λ*.

Allowing the transfer car and shuttles to operate simultaneously reduces the total processing time, while its effect on total waiting time depends on the number of shuttles and depth / width ratio. Specifically, the processing time of parallel task is the maximum of shuttle processing time and transfer car processing time. At the meantime, for systems with storage lanes that are not too deep (i.e., *dw* < dw*), the parallel policy increases the total waiting time due to a long travel distance of transfer car to pick up the waiting shuttles. This implies that the shuttles may always waiting for the service of transfer car, which resulting a higher utilization of transfer car and a longer waiting time of shuttles. For deep lane storage systems (i.e., *dw* > dw*), the situation reverses since the capacity of transfer car is sufficient so that the increase of shuttle waiting time is dominated by the reduction of total processing time. Therefore, the performance of parallel policy is better than that of sequential policy. In addition, increasing the number of shuttles can reduce the total waiting time since the expected travel distance of transfer car is shorter. Thus, the reduction of total processing time can offset the increase of total waiting time easier (i.e., dw* decreases).

### 5.3 Investigation of a real case

In this section, we estimate the performance of both sequential and parallel processing policies in a real case, which refers to a Nedcon system in UK [2]. The system consists of multiple tiers of multiple storage lanes with a layout as studied in our research. In each tier, there are 37 storage columns and 47 storage lanes at each side of the cross-aisle. As analyzing the real case, we should consider the effects of acceleration/deceleration of shuttles and the transfer car. Thus, the model has been adjusted to accommodate for acceleration/deceleration effects, which is referred to the work of Zou et al. [27]. And we also assume that *λ*_*r*_ = *λ*_*s*_. Other system parameters are described in Table 7.

**Table 7:** System parameters related to the real case.

Variable	Description	Value
*w*	Unit width per storage position	1.47m
*d*	Unit depth per storage position	0.9m
*t*_*t*_, *t*_*sh*_	Transfer car or shuttle loading/unloading time	3.5s; 6s
*v*_*t*_, *v*_*sh*_	Maximum velocity of transfer car and shuttle	1m/s
*a*_*t*_, *a*_*sh*_	Transfer car and shuttle acceleration/deceleration	0.3 m/s^2^; 0.4 m/s^2^

**Fig 11:**
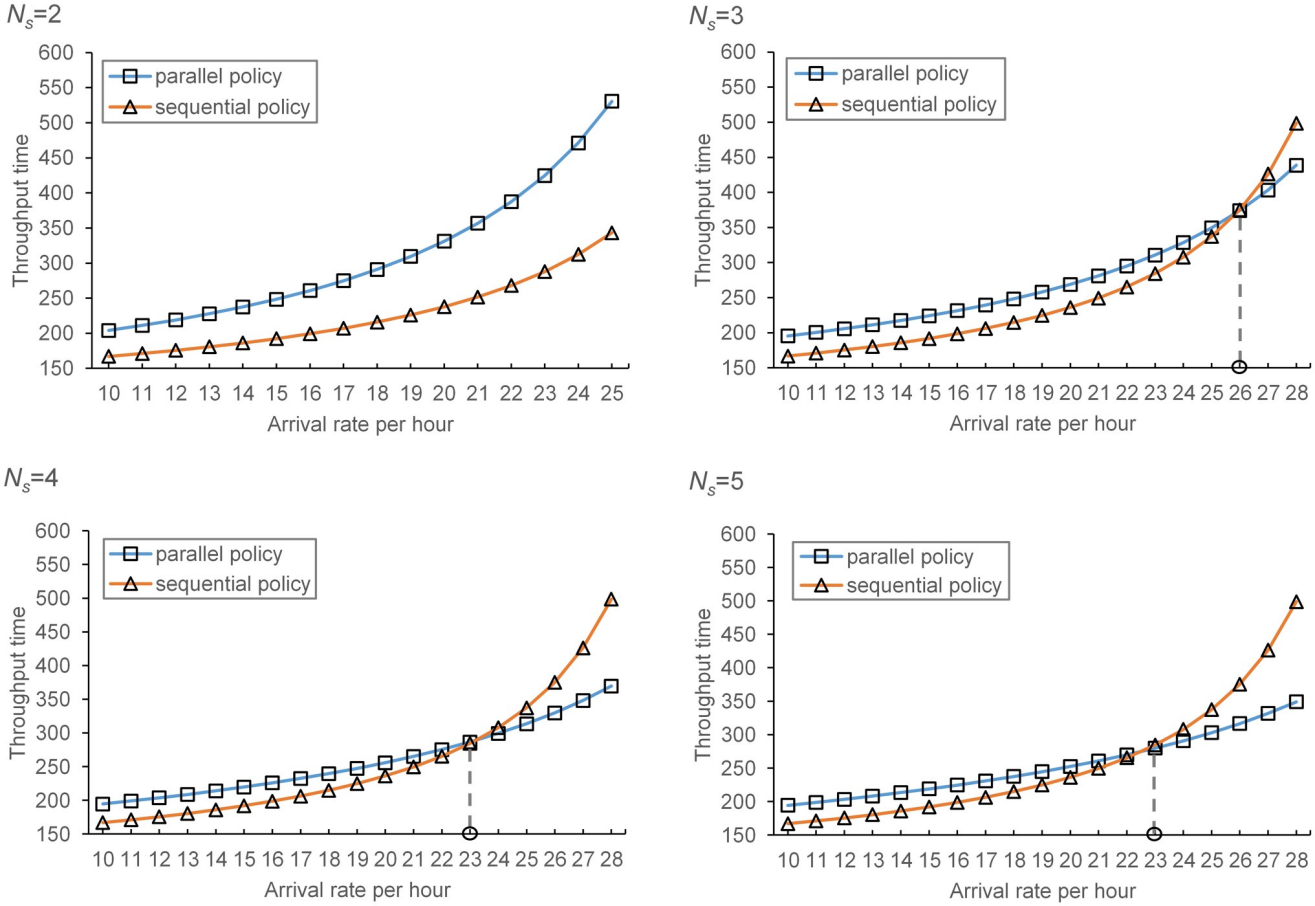
Comparison of parallel and sequential processing policies with different arrival rate.

As shown in previous discussion, the depth/width ratio, transaction arrival rate and number of shuttles may affect the performance of sequential and parallel processing policies. Thus, for the analysis of a real case, we first vary the transaction arrival rate, ranging from 10 to 28 with a step size of 1, and keep the other variables fixed to investigate potential improvement in throughput capacity as a result of adopting parallel processing policy with different resources utilizations. The results are showed in Fig 11.

With the increasing of transaction arrival rate, the increasing utilization of the transfer car increases the waiting time of shuttles for the service of the transfer car. Given the current configuration of the real system, the expected throughput capacity of sequential processing policy is larger than that of parallel processing when transaction arrival rate is relatively small. This may result from that when *λ* is small, the average waiting time of shuttles for the service of the transfer car is longer under parallel processing policy than that under sequential processing policy. For a large arrival rate, the situation reverses since the increase of shuttle waiting time, under parallel processing policy, is dominated by the reduction of total processing time. The intersection point between the curves of the sequential processing policy and the parallel processing policy shows the critical transaction arrival rate, below which the sequential processing policy outperforms the parallel processing policy. On the other hand, when the number of shuttles increases, the critical transaction arrival rate decreases since adding new shuttles may shorten the average shuttle waiting time for the service of transfer car. Specifically, the critical transaction arrival rate is about 26 per hour when *N*_*s*_ = 3, and about 23 when *N*_*s*_ = 4, 5. For the case of two shuttles, the critical transaction arrival rate is larger than 28 per hour, where the resource utilizations are higher than 95% and may not guarantee the conditions for convergence of the system. Thus, we eliminate the scenarios with transaction arrival rates larger than 26.

**Fig 12:**
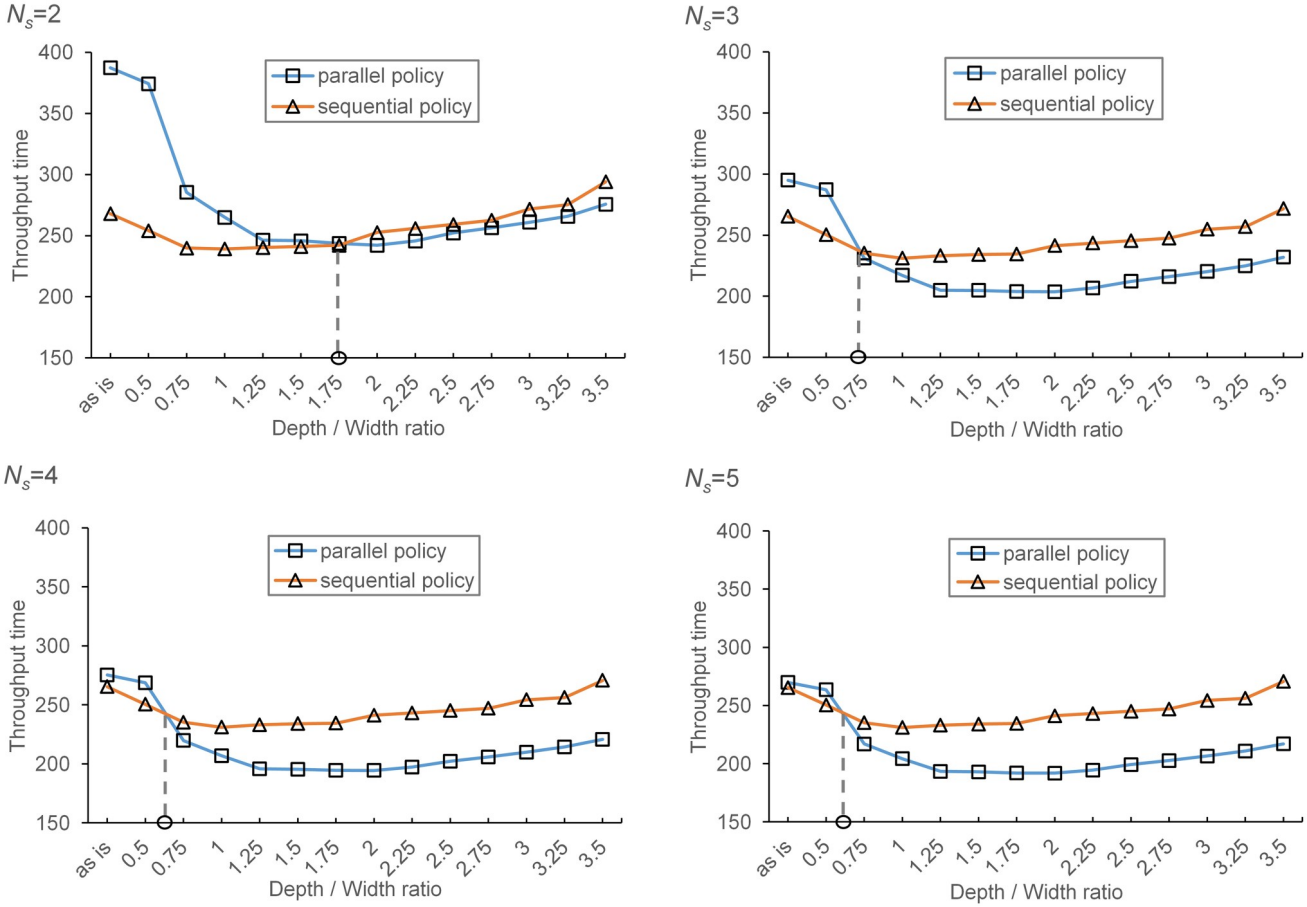
Comparison of parallel and sequential processing policies with different depth/width ratio.

For the analysis of the effects of tier configuration, we vary the depth/width ratio from 0.5 to 3.5 with a step size of 0.25, and keep the other variables fixed (the transaction arrival rate is 22 per hour). The results are provided in Fig 12.

The optimal depth/width ratio under parallel processing policy is 1.75, larger than that of sequential processing policy. This implies that, given the current system configurations, the maximum system throughput can be achieved when the depth/width ratio is 1.75 and the system throughput decreases as the depth/width ratio increase or decrease. And the curve is very flat at the optimal ratio point. As discussed in previous section, there exists a critical depth/width ratio, below which the sequential processing policy outperforms the parallel processing policy. And adding new shuttles also results a decreasing of the critical depth/width ratio. Specifically, the critical depth/width ratio is about 1.75 when *N*_*s*_ = 2, about 0.72 when *N*_*s*_ = 3, and about 0.625 when *N*_*s*_ = 4, 5.

Given the current configuration of the real system, the sequential processing policy outperforms the parallel processing policy. However, when the arrival rate of transactions becomes large (e.g., during COVID-19), the parallel processing policy should be considered. Our results also allow showing that the depth/width ratio have a significant impact on the difference in system performance between sequential and parallel processing policy. This implies the adoption of parallel processing policy may shorten system response time in the systems with deep storage lanes. Besides, despite the increase of investment cost, adding new shuttles may be a useful way to improve system performance since it will reduce the critical transaction arrival rate and encourage the transform of processing policy from sequential to parallel, which may further improve the system performance. For the system design, our results suggest that the optimal depth/width ratio should be used as a guiding factor.

**Fig 13:**
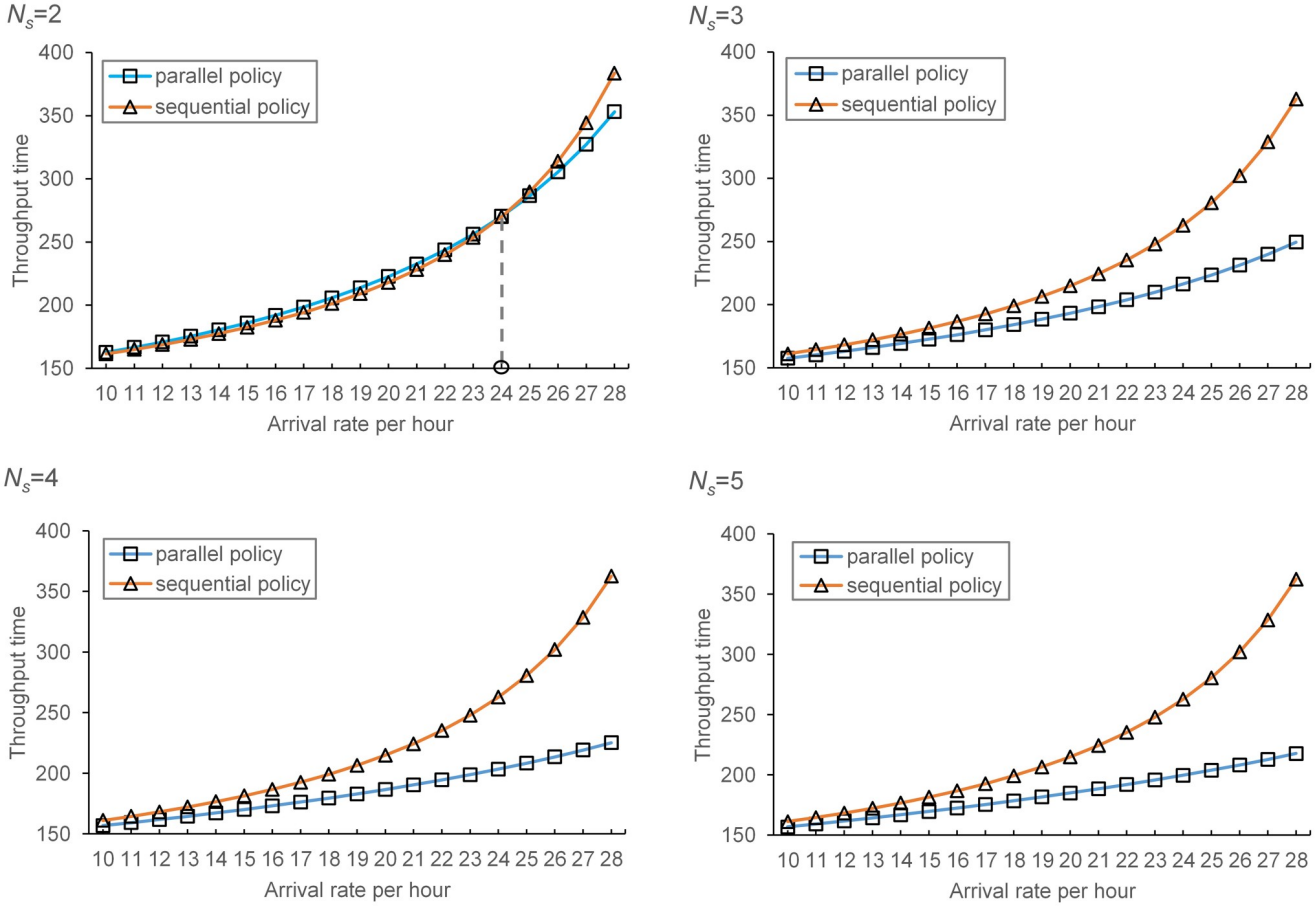
Comparison of parallel and sequential processing policies under the optimal depth/width ratio.

To better understand the system performance under different processing policies, we set the depth/width ratio at the optimal level (i.e., 1.75 for system under parallel processing policy and 1.25 for sequential processing policy), vary the transaction arrival rate, ranging from 10 to 28 with a step size of 1 and keep the other variables fixed. The results are provided in Fig 13.

When the number of shuttles is small (i.e., *N*_*s*_ = 2) and the arrival rate of transactions is relatively low (smaller than 24 per hour), the system throughput under sequential processing policy is better than that under parallel processing given the current system configurations and the optimal depth/width ratio (i.e., 1.75 for system under parallel processing policy and 1.25 for sequential processing policy). However, when adding new shuttles or the arrival rate of transactions becomes larger, the parallel policy outperforms the sequential processing policy. Besides, the advantage of the parallel processing policy increases with the increase of shuttle number and the transaction arrival rate. These results suggest that, considering the variety of customer demands, the parallel processing policy should be considered and the optimal depth/width ratio of parallel processing policy should be used as a guiding factor.

## 6 Conclusions and future works

The shuttle-based compact storage systems are becoming popular and adopted by many modern warehouses. Considering the variety of customer demands, it is important to improve the performance of such systems. Given its advantages in improving system performance, the parallel processing policy in shuttle-based compact storage systems need to be investigated. However, studies on this subject are rare. This study is one of the first to estimate the system performance of parallel processing policy in shuttle-based compact systems. Our contributions lie in both developing an analytical model and providing operational and design insights. Specifically, we mainly focus on the performance estimation of a single-tier of specialized shuttle-based compact storage system, in which the shuttles can only move within storage lanes and are transported along the cross-aisle by the transfer car, under parallel processing policy. The system is modelled as a multi-class semi-open queuing network with class switching, so that transfer car can perform other tasks during a retrieval transaction. Both storage and retrieval transactions are considered to capture the dynamic of shuttle routes and estimate the effect of different transactions on system throughput time. To capture the effect of simultaneously operations of the shuttles and the transfer car, we formulate a FJQN in which the transaction will be split into two parts, one is served by the shuttle and the other is served by the transfer car. Since exact solutions to the proposed semi-open queuing network are not available, a decomposition-based approach is developed to estimate the performance of the system. The analytical model is validated against simulations, the average errors for system response time, shuttle and transfer car utilization and external queue length are 6.32%, 2.93%, 2.38% and 10.81%, respectively.

We carry out a series of numerical experiments to compare the performance of sequential and parallel processing policies. The results show that the parallel processing policy outperforms the sequential processing policy in systems with deep storage lanes (which means the depth/width ratio of the system is large). Additionally, the advantage of the parallel processing policy increases with the increase of shuttle number, the depth/width ratio and the transaction arrival rate. Our results also show that there is a critical depth/width ratio, below which the system should follow the sequential processing policy. Otherwise, the parallel processing policy should be considered. We also investigate the performance of both sequential and parallel processing policies in a real case. Given the current configuration of the real system, the system response time of sequential processing policy is lower than that of parallel processing policy. However, when the transaction arrival rate becomes large, our results suggest benefits of adopting parallel processing policy. The results also show the critical point of transaction arrival rate and depth/width ratio under different shuttle numbers. Besides, the optimal depth/width ratio of the real system is 1.75 when parallel processing policy is used, which is independent of the shuttle number and the transaction arrival rate. When comparing the system performance of different processing policy under the optimal depth/width ratio (1.75 for parallel and 1.25 for sequential processing policy), the results show that the sequential processing policy only have advantages when there are two shuttles in a tier and the transaction arrival rate is small (smaller than 24 per hour). This suggests a potential improvement in system performance achieved by the adoption of parallel processing policy considering the variety of customer demand.

This study provides some useful managerial implications and warehouse design insights. However, there is nevertheless a set of limitations. First, the proposed model is only applied on only one real system. Thus, the findings, such as the optimal and critical depth/width ratio and the potential improvement in system performance achieved by adopting parallel processing policy, may not be applicable to other warehouses with different system configurations. Second, in order to develop a tractable model, some assumptions are made in this study, such as random storage policy, POSC dwell point policy, FCFS scheduling policy and so on, all of which could be relaxed. therefore, for future research, it is interesting to consider the effect of different storage assignment policies, different dwell point policies, different transaction scheduling policies, different shuttle assignment rules and the blocking effects. Additionally, it would be interesting to investigate the system performance with transactions requiring more than one unit load and considering both single- and dual-command cycles. On the other hand, future research would include applying the proposed model on other systems where resources work simultaneously and developing more accurate and robust modeling approaches.

## Supporting information

S1 File Details on the component of transition matrix *Q* of FJQN. (PDF)

S2 File Details on the solution approach for reduced network with a single server. (PDF)

S3 File Details of simulation models. (PDF)
