# Supplementary material for: Modeling and performance analysis of shuttle-based compact storage systems under parallel processing policy
Source: PLoS One. 2021 Nov 15;16(11):e0259773. doi: 10.1371/journal.pone.0259773 (PMC8592453; doi:10.1371/journal.pone.0259773)
Supplement: S1 File — (PDF) [file pone.0259773.s001.pdf]

Details on the component of transition matrix $\boldsymbol{Q}$ of FJQN.

The load-dependent service rate of closed FJQN can be derived from the follow equations:

$$\mu_f(N_f)=\sum_{N_{ws}=1}^{N_f}\frac{\pi(N_{ws},0)}{E(T_2)}+\sum_{N_{ws}=0}^{N_f-1}\frac{\pi(N_{ws},1)}{E(T_1)}$$

$$\begin{cases}\boldsymbol{\pi Q}=0\\ \boldsymbol{\pi e}=1\end{cases}$$

To this end, we first approximate the general distributions of the service time at nodes 1 and 2 by Erlang-k distribution, where k denotes the number of exponential phases and $k=[1/cv^2]$. The mean service time at each phase, $\mu^{-1}=E(T)/k$. Let $\boldsymbol{\alpha}$ be the initial state probability, $\boldsymbol{T}$ be the phase transition matrix and $\boldsymbol{T^0}$ be the absorbing rate matrix. In equations reported below, the subscript s represents node 1, and c represents node 2. Thus, we can obtain:

$\boldsymbol{\alpha}_s=[1,0,\cdots,0]_{1\times k_s}$, $\boldsymbol{\alpha}_c=[1,0,\cdots,0]_{1\times k_c}$

$$\boldsymbol{T}_s=\begin{bmatrix}-\mu_s & \mu_s & 0 & \cdots & 0 & 0\\ 0 & -\mu_s & \mu_s & \cdots & 0 & 0\\ \vdots & \vdots & \vdots & \ddots & \vdots & \vdots\\ 0 & 0 & 0 & \cdots & -\mu_s & \mu_s\\ 0 & 0 & 0 & \cdots & 0 & -\mu_s\end{bmatrix}_{k_s\times k_s},\ \boldsymbol{T}_c=\begin{bmatrix}-\mu_c & \mu_c & 0 & \cdots & 0 & 0\\ 0 & -\mu_c & \mu_c & \cdots & 0 & 0\\ \vdots & \vdots & \vdots & \ddots & \vdots & \vdots\\ 0 & 0 & 0 & \cdots & -\mu_c & \mu_c\\ 0 & 0 & 0 & \cdots & 0 & -\mu_c\end{bmatrix}_{k_c\times k_c}$$

$$\boldsymbol{T}_s^0=\begin{bmatrix}0\\0\\ \vdots\\0\\ \mu_s\end{bmatrix}_{k_s\times 1},\ \boldsymbol{T}_c^0=\begin{bmatrix}0\\0\\ \vdots\\0\\ \mu_c\end{bmatrix}_{k_c\times 1}$$

The state space variable can be defined as $st_q=(N_{ws},N_{wt},i,j)$, where N_{ws} is the number of waiting shuttles in the join queue Q_{sh}, N_{wt} is the number of waiting transfer cars in the join queue Q_t and i,j denote the current phase of service process of shuttle and transfer car, respectively. Since there is only one transfer car in the system, i.e., $N_{wt}=0,1$, we denote the vector of the state probabilities with $N_{wt}=0$ as $\boldsymbol{\pi}_0$ and $N_{wt}=1$ as $\boldsymbol{\pi}_1$. The transition matrix $\boldsymbol{Q}$, therefore, consists of four components: the transition rates from the state of $\boldsymbol{\pi}_0$ to the state of $\boldsymbol{\pi}_0$, $\boldsymbol{\pi}_0$ to $\boldsymbol{\pi}_1$, $\boldsymbol{\pi}_1$ to $\boldsymbol{\pi}_0$ and $\boldsymbol{\pi}_1$ to $\boldsymbol{\pi}_1$, which are represented by $\boldsymbol{R}_{00}$, $\boldsymbol{R}_{01}$, $\boldsymbol{R}_{10}$ and $\boldsymbol{R}_{11}$, respectively. Hence, we can obtain:

$$\boldsymbol{Q}=\begin{bmatrix}\boldsymbol{R}_{00} & \boldsymbol{R}_{01}\\ \boldsymbol{R}_{10} & \boldsymbol{R}_{11}\end{bmatrix}$$

$$\boldsymbol{R}_{00}=\begin{bmatrix}\boldsymbol{T}_c\oplus\boldsymbol{T}_s & \boldsymbol{I}_c\otimes\boldsymbol{T}_s^0\boldsymbol{\alpha}_s & & & & \\ \boldsymbol{T}_c^0\boldsymbol{\alpha}_c\otimes\boldsymbol{I}_s & \boldsymbol{T}_c\oplus\boldsymbol{T}_s-\boldsymbol{T}_c^1 & \boldsymbol{I}_c\otimes\boldsymbol{T}_s^0\boldsymbol{\alpha}_s & & & \\ & \boldsymbol{T}_c^0\boldsymbol{\alpha}_c\otimes\boldsymbol{I}_s & \boldsymbol{T}_c\oplus\boldsymbol{T}_s-\boldsymbol{T}_c^1 & & & \\ & & \ddots & \ddots & \ddots & \\ & & & \boldsymbol{T}_c^0\boldsymbol{\alpha}_c\otimes\boldsymbol{I}_s & \boldsymbol{T}_c\oplus\boldsymbol{T}_s-\boldsymbol{T}_c^1 & \boldsymbol{I}_c\otimes\boldsymbol{T}_s^0\\ & & & & \boldsymbol{T}_c^0\boldsymbol{\alpha}_c\otimes\boldsymbol{\alpha}_s & \boldsymbol{T}_c\end{bmatrix}$$

$$\boldsymbol{R}_{01}=\begin{bmatrix}\boldsymbol{T}_c^0\otimes\boldsymbol{I}_s & & & \\ & \boldsymbol{T}_c^0\otimes\boldsymbol{I}_s & & \\ & & \ddots & \\ & & & \boldsymbol{T}_c^0\otimes\boldsymbol{I}_s\\ 0 & 0 & \cdots & 0\end{bmatrix}$$

$$
\boldsymbol{R}_{10} = \begin{bmatrix} \boldsymbol{\alpha}_c \otimes \boldsymbol{T}_s^0 \boldsymbol{\alpha}_s & & & & 0 \\ & \boldsymbol{\alpha}_c \otimes \boldsymbol{T}_s^0 \boldsymbol{\alpha}_s & & & 0 \\ & & \ddots & & \vdots \\ & & & \boldsymbol{\alpha}_c \otimes \boldsymbol{T}_s^0 \boldsymbol{\alpha}_s & 0 \end{bmatrix}
$$

$$
\boldsymbol{R}_{11} = \begin{bmatrix} \boldsymbol{T}_s - \boldsymbol{T}_s^1 & \boldsymbol{T}_s^0 \boldsymbol{\alpha}_s & & & & \\ & \boldsymbol{T}_s - \boldsymbol{T}_s^1 & \boldsymbol{T}_s^0 \boldsymbol{\alpha}_s & & & \\ & \ddots & \ddots & \ddots & & \\ & & \boldsymbol{T}_s - \boldsymbol{T}_s^1 & \boldsymbol{T}_s^0 \boldsymbol{\alpha}_s & & \\ & & & \boldsymbol{T}_s - \boldsymbol{T}_s^1 & \boldsymbol{T}_s^0 \boldsymbol{\alpha}_s & \\ & & & & \boldsymbol{T}_s & \end{bmatrix}
$$

where $\boldsymbol{I}_s$ and $\boldsymbol{I}_c$ are identity matrices of size $k_s \times k_s$ and $k_c \times k_c$, respectively. $\oplus$ is Kronecker sum and $\otimes$ is Kronecker product, and

$$
\boldsymbol{T}_s^1 = \begin{bmatrix} 0 & & & & \\ & 0 & & & \\ & & \ddots & & \\ & & & 0 & \\ & & & & \mu_s \end{bmatrix}_{k_s \times k_s}, \quad \boldsymbol{T}_c^1 = \begin{bmatrix} 0 & & & & \\ & 0 & & & \\ & & \ddots & & \\ & & & 0 & \\ & & & & \mu_c \end{bmatrix}_{k_c \times k_c} \otimes \boldsymbol{I}_s
$$

Thus, we can obtain the state probabilities $\boldsymbol{\pi}$ and the load-dependent service rate of FJQN, $\mu_f(N_f)$.
