# Supplementary material for: Modeling and performance analysis of shuttle-based compact storage systems under parallel processing policy
Source: PLoS One. 2021 Nov 15;16(11):e0259773. doi: 10.1371/journal.pone.0259773 (PMC8592453; doi:10.1371/journal.pone.0259773)
Supplement: S2 File — (PDF) [file pone.0259773.s002.pdf]

Details on the solution approach for reduced network with a single server.

The steady state probabilities for the CTMC are obtained using flow rate balance equations. According to the birth–death process of the reduced network, we can get:

$$\begin{cases} \lambda\pi(-N_s) = \mu(1)\pi(-N_s+1) \\ \lambda\pi(-N_s+1) = \mu(2)\pi(-N_s+2) \\ \vdots \\ \lambda\pi(-2) = \mu(N_s-1)\pi(-1) \\ \lambda\pi(-1) = \mu(N_s)\pi(0) \\ \lambda\pi(0) = \mu(N_s)\pi(1) \\ \lambda\pi(1) = \mu(N_s)\pi(2) \\ \vdots \end{cases} \quad (S2.1)$$

Therefore:

$$\begin{cases} \pi(-N_s+1) = \frac{\lambda\pi(-N_s)}{\mu(1)} \\ \pi(-N_s+2) = \frac{\lambda\pi(-N_s+1)}{\mu(2)} = \frac{\lambda\pi(-N_s)}{\mu(1)}\frac{\lambda}{\mu(2)} = \frac{\lambda^2}{\mu(1)\mu(2)}\pi(-N_s) \\ \vdots \\ \pi(-1) = \frac{\lambda^{N_s-1}}{\mu(1)\mu(2)\cdots\mu(N_s-1)}\pi(-N_s) \\ \pi(0) = \frac{\lambda^{N_s}}{\mu(1)\mu(2)\cdots\mu(N_s-1)\mu(N_s)}\pi(-N_s) \\ \pi(1) = \frac{\lambda}{\mu(N_s)}\pi(0) = \frac{\lambda}{\mu(N_s)}\cdot\frac{\lambda^{N_s}}{\mu(1)\mu(2)\cdots\mu(N_s-1)\mu(N_s)}\pi(-N_s) \\ \pi(2) = (\frac{\lambda}{\mu(N_s)})^2\cdot\frac{\lambda^{N_s}}{\mu(1)\mu(2)\cdots\mu(N_s-1)\mu(N_s)}\pi(-N_s) \\ \vdots \end{cases} \quad (S2.2)$$

Thus, by generalizing (S2.2), we get:

$$\pi(x) = \begin{cases} \pi(-N_s)\prod_{i=-N_s+1}^{x}\frac{\lambda}{\mu(N_s+i)}, -N_s < x \le 0 \\ \pi(-N_s)\prod_{i=-N_s+1}^{0}\frac{\lambda}{\mu(N_s+i)}\cdot(\frac{\lambda}{\mu(N_s)})^x, x > 0 \end{cases} \quad (S2.3)$$

Besides, according to the law of total probability, we get:

$$\sum_{i=-N_s}^{\infty}\pi(i) = 1 \quad (S2.4)$$

$$\sum_{i=-N_s}^{-1}\pi(i) + \sum_{i=0}^{\infty}\pi(i) = 1 \quad (S2.5)$$

and we know:

$$\sum_{i=0}^{\infty}\pi(i) = \pi(-N_s)\prod_{j=-N_s+1}^{0}\frac{\lambda}{\mu(N_s+j)}\sum_{i=0}^{\infty}\left(\frac{\lambda}{\mu(N_s)}\right)^i$$

$$= \pi(-N_s)\prod_{j=-N_s+1}^{0}\frac{\lambda}{\mu(N_s+j)}\cdot(\frac{1}{1-\frac{\lambda}{\mu(N_s)}}) \quad (S2.6)$$

Combining (S2.5) and (S2.6), and let $k = N_s + j$, we get:

$$\pi(-N_s) = \frac{1}{1+\sum_{i=1}^{N_s-1}\prod_{k=1}^{i}\frac{\lambda}{\mu(k)}+(\frac{\mu(N_s)}{\mu(N_s)-\lambda})\prod_{k=1}^{N_s}\frac{\lambda}{\mu(k)}} \quad (S2.7)$$
