# Supplementary material for: Modeling and performance analysis of shuttle-based compact storage systems under parallel processing policy
Source: PLoS One. 2021 Nov 15;16(11):e0259773. doi: 10.1371/journal.pone.0259773 (PMC8592453; doi:10.1371/journal.pone.0259773)
Supplement: S3 File — (PDF) [file pone.0259773.s003.pdf]

**Details of simulation models**

To validate the analytical model, we build a simulation model using Arena 14.0 software. In our simulation model, specifically, the arrival of both storage and retrieval transactions are assumed to follow a Poisson distribution and the random storage policy is used. Like in the analytical model, the movements of shuttle and transfer car are simultaneously, and the shuttle blocking effects within a storage lane is ignored. However, in regards to the modeling of service times, a discrete space is assumed in our simulation model, while a continuous space is considered in the analytical model. The uniform distribution is used to model the service times $t_{sh1}$ and $t_{t1}$, the lognormal distribution to model $t_{sh2}$ and $t_{t2}$, and a constant to model $t_{sh3}$ and $t_{t3}$. Fig S3.1 shows the flowchart of the simulation model.

The followings are the main events and processes in the simulation model:

1. Arrival of transactions: the arrival processes of storage and retrieval transactions follow a Poisson distribution with arrival rate $\lambda_s$ and $\lambda_r$, respectively.
2. Simultaneously operations of shuttles and the transfer car: when a transaction is assigned to a shuttle, a request is made by the shuttle for transfer car simultaneously. The simulation model captures this process by a "split" module, where the transaction is split into two parts, one is served by the shuttle and the other is served by the transfer car.
3. Synchronization of shuttles and the transfer car: if the shuttle reaches the first bay of its lane first, it will wait for the transfer car. Otherwise, the transfer car will wait for the shuttle at the intersection point of the shuttle's lane and the cross-aisle. The simulation model captures this by a "match" modular that contains two queues. The shuttle waits for the transfer car in one queue and the transfer car waits for the shuttle in another. When both are in position, they will join into a single transaction.
4. Joint movement of shuttles and the transfer car: After the synchronization of the shuttle and the transfer car, the transfer car will move the shuttle to its destination lane and releases it. The transfer car is released after this operation.
5. Departure of the transaction: The transaction is finished when the shuttle releases the load at the I/O point or the destination storage position. The shuttle is released after this operation.

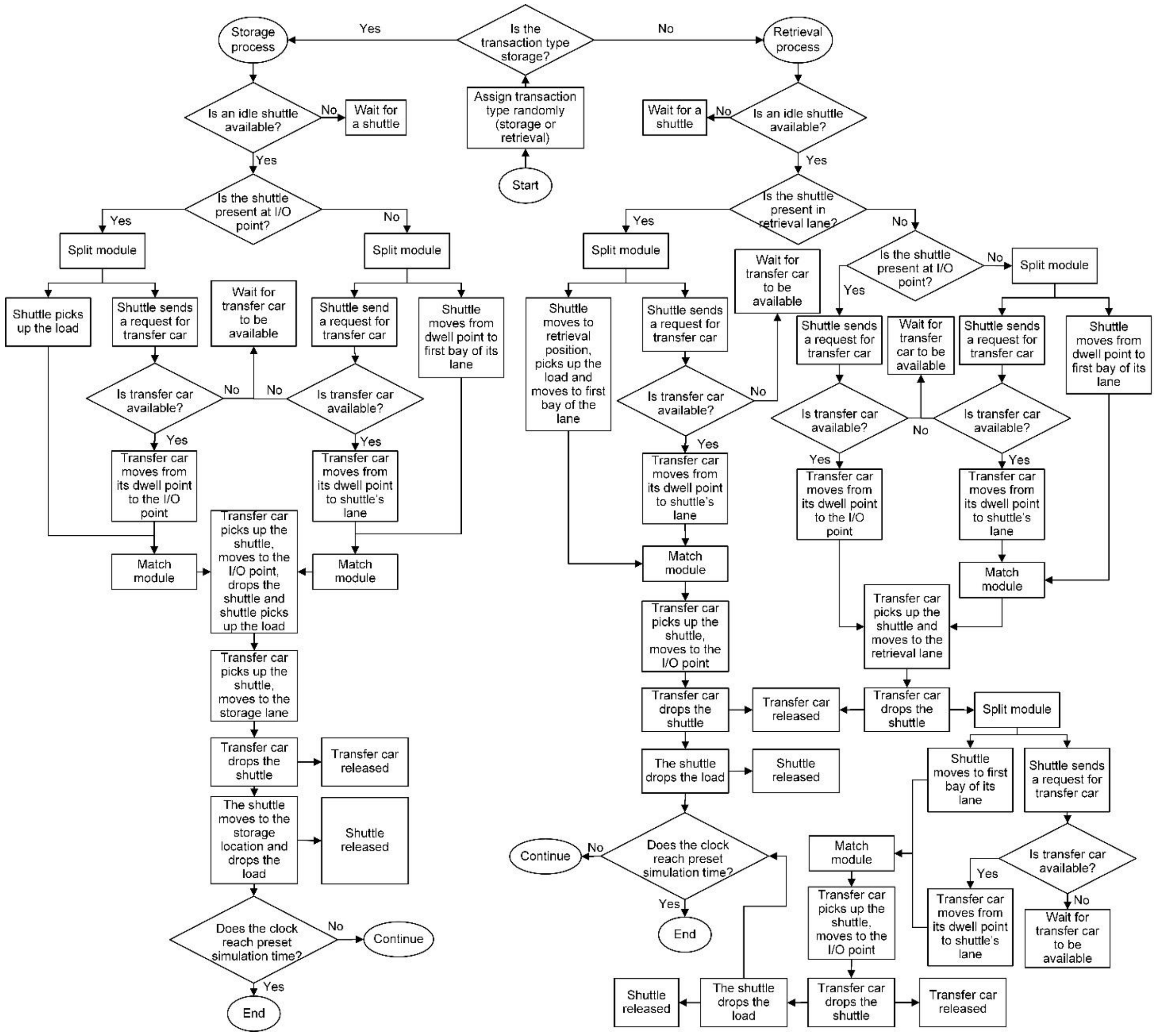


**Fig S3.1 Flowchart of simulation model**
